# Pacmanvirus isolated from the Lost City hydrothermal field extends the concept of transpoviron beyond the family *Mimiviridae*

**DOI:** 10.1093/ismejo/wraf002

**Published:** 2025-01-10

**Authors:** Sébastien Santini, Audrey Lartigue, Jean-Marie Alempic, Yohann Couté, Lucid Belmudes, William J Brazelton, Susan Q Lang, Jean-Michel Claverie, Matthieu Legendre, Chantal Abergel

**Affiliations:** Information Génomique & Structurale, Unité Mixte de Recherche 7256, Aix-Marseille University, Centre National de la Recherche Scientifique, IMM, IM2B, IOM, 13288, Marseille Cedex 9, France; Information Génomique & Structurale, Unité Mixte de Recherche 7256, Aix-Marseille University, Centre National de la Recherche Scientifique, IMM, IM2B, IOM, 13288, Marseille Cedex 9, France; Information Génomique & Structurale, Unité Mixte de Recherche 7256, Aix-Marseille University, Centre National de la Recherche Scientifique, IMM, IM2B, IOM, 13288, Marseille Cedex 9, France; Univ. Grenoble Alpes, INSERM, CEA, UA13 BGE, CNRS, CEA, FR2048, 38000 Grenoble, France; Univ. Grenoble Alpes, INSERM, CEA, UA13 BGE, CNRS, CEA, FR2048, 38000 Grenoble, France; School of Biological Sciences, University of Utah, Salt Lake City, United States; Department of Geology and Geophysics, Woods Hole Oceanographic Institution, Woods Hole, MA United States; Information Génomique & Structurale, Unité Mixte de Recherche 7256, Aix-Marseille University, Centre National de la Recherche Scientifique, IMM, IM2B, IOM, 13288, Marseille Cedex 9, France; Information Génomique & Structurale, Unité Mixte de Recherche 7256, Aix-Marseille University, Centre National de la Recherche Scientifique, IMM, IM2B, IOM, 13288, Marseille Cedex 9, France; Information Génomique & Structurale, Unité Mixte de Recherche 7256, Aix-Marseille University, Centre National de la Recherche Scientifique, IMM, IM2B, IOM, 13288, Marseille Cedex 9, France

**Keywords:** pacmanvirus, *Asfarviridae*, mobile genetic elements, Lost City

## Abstract

The microbial sampling of submarine hydrothermal vents remains challenging, with even fewer studies focused on viruses. Here we report what is to our knowledge the first isolation of a eukaryotic virus from the Lost City hydrothermal field, by co-culture with the laboratory host *Acanthamoeba castellanii*. This virus, named pacmanvirus lostcity, is closely related to previously isolated pacmanviruses (strains A23 and S19), clustering in a divergent clade within the long-established family *Asfarviridae.* The icosahedral particles of this virus are 200 nm in diameter, with an electron-dense core surrounded by an inner membrane. The viral genome of 395 708 bp (33% G + C) has been predicted to encode 473 proteins. However, besides these standard properties, pacmanvirus lostcity was found to be associated with a new type of selfish genetic element, 7 kb in length, whose architecture and gene content are reminiscent of those of transpovirons, hitherto specific to the family *Mimiviridae*. As in previously described transpovirons, this selfishg genetic element propagates as an episome within its host virus particles and exhibits partial recombination with its genome. In addition, an unrelated episome with a length of 2 kb was also found to be associated with pacmanvirus lostcity. Together, the transpoviron and the 2-kb episome might participate in exchanges between pacmanviruses and other DNA virus families. It remains to be elucidated if the presence of these mobile genetic elements is restricted to pacmanviruses or was simply overlooked in other members of the *Asfarviridae*.

## Introduction

Since the discovery of mimivirus [1], amoeba coculture has been used to isolate giant viruses from a variety of soils and aquatic environments ranging from cryosols [2] to hot springs [3] and even across ages [4–6]. However, there are still many difficult to access places on our planet that remain unexplored. The Lost City hydrothermal field is one of these. It was discovered in 2000, 15 km off the Mid-Atlantic Ridge, at a depth of 700–800 m [7]. The temperatures observed in Lost City venting fluids vary between ~7°C and 90°C [8] and are compatible with life. The high pH (from 9 to 11) and the high concentrations of hydrogen and methane are characteristic of the serpentinization processes associated with the uplift of the host mantle rocks. Similar serpentinization-associated reactions on the early Earth could have provided favorable conditions for the emergence of life [9]. Various species ranging from macrofaunal communities to microorganisms have been observed [7, 8, 10], and traces of DNA of possible viral origin were found in the biofilms growing around the carbonate chimneys of the site [11]. Although the presence of viruses near hydrothermal vents has been previously reported [12–15], as well as that of members of the phylum *Nucleocytoviricota* [16] in deep-sea sediments [17], the isolation and characterization of viruses at these sites is still in its infancy.

**Figure 1:**
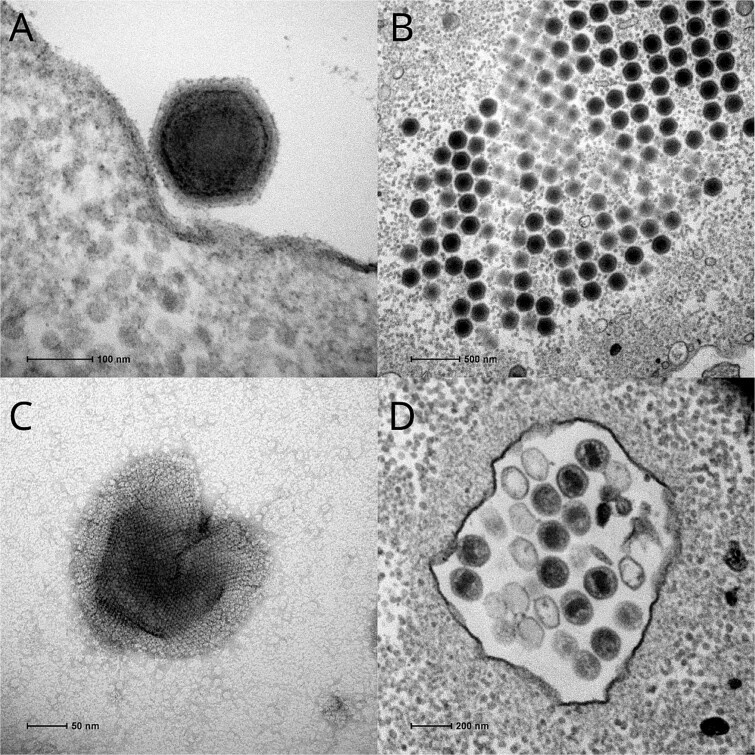
Transmission electron microscopy (TEM) images of pacmanvirus lostcity*.* Ultrathin section of cells infected by pacmanvirus lostcity observed by negative staining TEM of (A) an isolated virion, (B) mature virions at 6 h postinfection, (C) negative staining TEM image of full virion losing its external proteinaceous layer, and (D) virions in infected cells (30 minutes postinfection), some presenting an inner rectangular-shaped core, some being empty.

**Figure 2:**
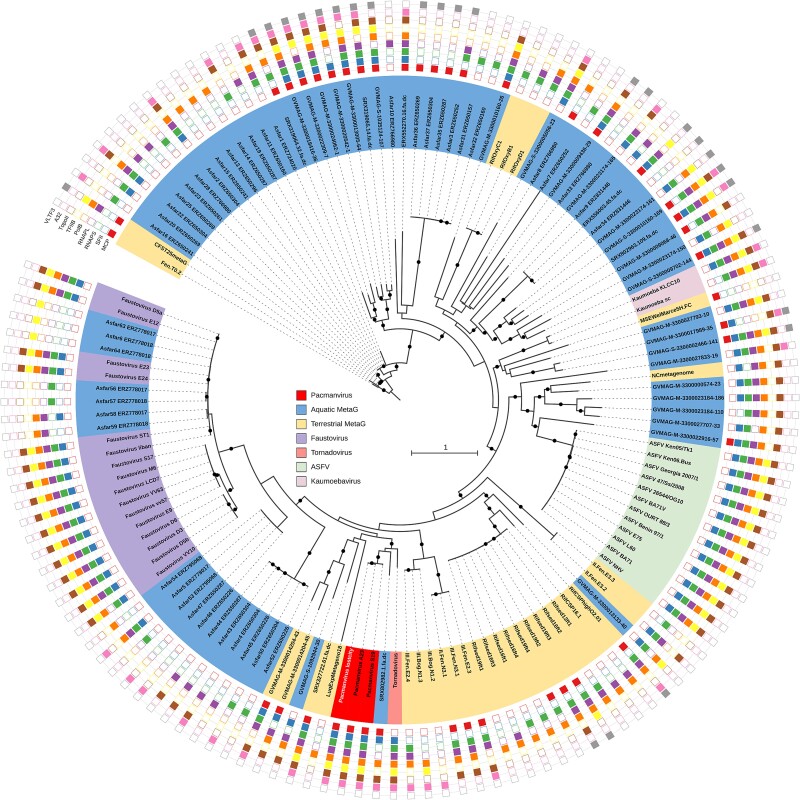
Phylogeny of pacmanviruses among the extended family *Asfarviridae*. The tree was obtained using data detailed in Table S2 and the method described in the material and method section. For each of the 9 possible markers (NCLDV major capsid protein, DEAD/SNF2-like helicase, DNA-directed RNA polymerase beta subunit, DNA-directed RNA polymerase alpha subunit, DNA polymerase family B, transcription initiation factor IIB, DNA topoisomerase II, packaging ATPase, poxvirus viral late transcription factor 3) used, an empty square indicates that the marker was not found by ncldv_markersearch. Only bootstraps greater than 90% are reported with a black circle. The scale bar and the color legend of the different datasets or organisms are indicated in the center of the tree.

The phylum *Nucleocytoviricota* comprises large eukaryote-infecting DNA viruses classified in separate orders (*Algavirales*, *Imitervirales*, *Pimascovirales*, *Asfuvirales*, *Chitovirales*) [18] and additional proposed families such as *Molliviridae*, *Faustoviridae*, *Kaumoebaviridae*, and *Pacmanviridae*[19]. The pacmanvirus takes its name from the broken appearance of some of its particles when observed by transmission electron microscopy [19], (Fig. 1C). The first two strains (A23 and S19) were isolated from Algerian samples by co-culture with *Acanthamoeba castellanii* [19, 20]. The pacmanviruses form a distinct clade within the highly diverse order *Asfuvirales*, which encompasses different strains of African swine fever virus (ASFV), the distantly related faustoviruses and kaumoebavirus, and a large number of aquatic and terrestrial metagenomes (Fig. 2).

Among the phylum *Nucleocytoviricota*, only members of the family *Mimiviridae* [21] and *Poxviridae* [22] are associated with selfish genetic elements, virophages [23] and transpovirons [21, 24].

**Figure 3:**
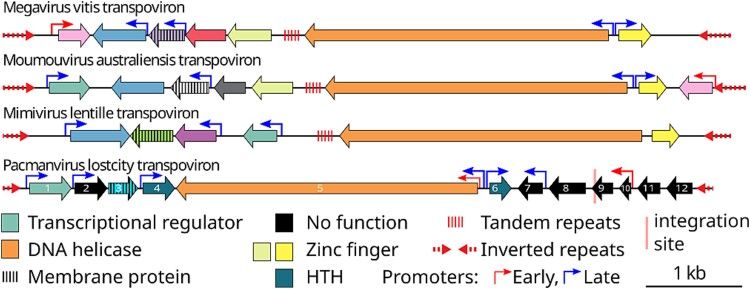
Gene content and detailed genomic structure of pltv compared to other known transpovirons*.* Early and late promoters correspond to the ones of the giant virus hosting the transpoviron. In pacmanvirus lostcity they were defined as AAAATTGA and TATATA respectively.

Transpovirons are linear DNA molecules of about 7 kb that share the same genomic organization and protein-coding gene content [21, 24] (Fig. 3). All known transpovirons encode a DNA helicase, predicted nucleic acid*–*interacting proteins, and a probable transmembrane protein [24]. These molecules all present an internal tandem repeat and inverted repeats at their extremities (Fig. 3).

In this report we describe a new pacmanvirus isolated from the Lost City hydrothermal vent field. Among other specific features, this new strain (called “pacmanvirus lostcity”) was found to be unexpectedly associated with a transpoviron, thus extending the known range of propagation of these genetic elements well beyond the family *Mimiviridae*.

## Materials and methods

### Viral culture, isolation, cloning, and purification of pacmanvirus and its transpoviron

A chimney subsample (LC02854) was collected from the “IMAX” flange at Marker 2 as a “grab sample” with remotely operated underwater vehicle *Jason*’s manipulator arm during the 2018 Lost City expedition. At the time of sampling, the IMAX flange at Marker 2 was venting fluids with a maximum temperature of approximately 58°C. Shipboard, the chimney subsample was submerged in a rice medium (prepared by cooking 4 grains of rice in 1 l artificial seawater) and stored at 4°C until shipment to the Brazelton Laboratory in Utah, where it was accidentally stored at −80°C. The sample was subsequently shipped to the Laboratoire Information Génomique et Structurale in France on dry ice for further analyses. Virus isolation and cloning were performed using our previously described protocol [25].

Briefly, 25 ml of 40-mM Tris pH 7.5 buffer was added to a fragment of ~25 g of the white chimney subsample with seawater and vortexed at room temperature. After decanting for 3 minutes, the supernatant was taken up and centrifuged at 10 000 g for 1 hour. The pellet was then resuspended in 40 mM Tris pH 7.5 with a cocktail of antibiotics (ampicillin 100 μg/ml, chloramphenicol 34 μg/ml, kanamycin 20 μg/ml) and Fungizone (amphotericin B) (4%). This preparation was then deposited one drop at a time onto two 15-cm–diameter Petri dishes (Sarsted 82.1184.500), one previously seeded with 500 cells/cm^2^ regular *Acanthamoeba castellanii* and the other with *A. castellanii* cells previously adapted to Fungizone (1.25%).

Cytopathic effects were detected, and polymerase chain reaction (PCR) amplifications using the Terra PCR Direct Polymerase Mix (Takara Bio Europe SAS, Saint-Germain-en-Laye, France) were performed using a set of in-house–designed family-specific primers. Amplicons were then sequenced and blast searches against our in-house viral database were performed. Pacmanvirus lostcity was then amplified in T-175 tissue-culture flasks plated with fresh *A. castellanii* cells and purified using sucrose gradients. For cloning, the virus at a multiplicity of infection of 10 was added to cells cultured in PPYG medium in one well of a 12-well plate, and after 1 hour the excess virions were removed by several rounds of washing. The cells were then recovered by gently scraping the well, and a serial dilution was performed in the next three wells by mixing 200 μl of the previous well with 500 μl of PPYG. Drops of 0.5 μl of the last dilution were recovered and observed by light microscopy to verify that there were fewer than two cells. The 0.5-μl droplets were then distributed in each well of a 24-well culture plate. One thousand uninfected *A. castellanii* cells in 500 μl of PPYG were added to the wells seeded with a single cell and monitored for cell death. The corresponding viral clones were recovered and amplified as described elsewhere [25] before purification, DNA extraction, proteome analysis, and cell cycle characterization by electron microscopy.

### Cell cycle and imaging

For transmission electron microscopy imaging, after synchronous infection, *A.* castellanii–infected cells were recovered along the infectious cycle, fixed by adding an equal volume of phosphate-buffered saline (PBS) with 2% glutaraldehyde, and incubated for 20 minutes at room temperature. Cells were pelleted for 20 minutes at 5000 *g*. The pellet was resuspended in 1 ml of PBS with 1% glutaraldehyde, incubated for at least 1 hour at 4°C, and washed twice in PBS before coating in agarose and embedding in Epon resin. Each pellet was mixed with 2% low-melting agarose and centrifuged to obtain small flanges of approximately 1 mm^3^ containing the sample coated with agarose. These samples were then embedded in Epon resin using a standard method: 1-hour fixation in 1% osmium tetroxide, dehydration in increasing ethanol concentrations (50%, 70% including uranyl acetate 2%, 90%, and 100% ethanol), and embedding in Epon-812. Ultrathin sections of 70 nm were poststained with 4% uranyl acetate and lead citrate and observed using an FEI Tecnai G2 operating at 200 kV. After a sucrose gradient or 0.45-nm filtration was performed to purify the virions, negative-staining transmission electron microscopy images of the purified virions were also produced.

### Genome sequencing, assembly, and polishing

Genomic DNA was recovered from purified virus particles from two clones using a PureLink genomic extraction minikit according to the manufacturer’s recommendations. The transpoviron DNA was also extracted and purified from an agarose gel. All of these samples were sequenced using Illumina technology on a NovaSeq6000 by Novogene. A nonclonal population was sequenced using MiSeq and the Oxford Nanopore Technology sequencer PromethION (ONT PromethION). The quality control using FastQC revealed a quality score > 30 over all bases for all Illumina reads and no sequencing contaminants. Nevertheless, non-ambiguous host genome fragments as well as the entire mitochondrial host genome were found and removed during the assembly process.

In a first attempt, each clone dataset was assembled independently using SPAdes 3.13.1 [26]. A two-way assembly was necessary to obtain the different genome models. The Illumina-driven assembly was achieved using SPAdes 3.13.1 on both Illumina and ONT PromethION reads with the *—careful* option. Illumina reads were mapped back to the genome model obtained using bowtie 2.3.4.1 [27] with the *—very-sensitive* option. ONT PromethION reads were mapped using minimap 2.17 [28] with its default parameters. All mapped reads were used with pilon 1.2.3 [29] and default parameters to polish this genome model. The ONT PromethION–driven assembly was achieved on ONT PromethION data with Flye 2.7.1 [30] and the following options: *-g 500 k —plasmids —meta*. Illumina reads were mapped to the genome model obtained using bowtie 2.3.4.1 with default parameters and submitted to pilon 1.2.3 with the option *—fix all novel*. All reads were mapped to the final genome models as described above.

### Genome annotation

GeneMarkS 4.32 [31] and the *—virus* option were used to predict 473 genes in the viral genome, 12 genes in the transpoviron sequence, and 4 genes in the 2-kb episome sequence. Corresponding protein sequences were submitted to blastp [32] against the NCBI nr database with a E-value threshold of 1e^−5^. The viral proteins that did not match any known pacmanvirus strain protein were submitted to a tblastn search (E-value threshold of 1e^−5^) against these genomes. A domain search was performed against Pfam 32.0, TIGRFAM 15.0, SMART 7.1, ProDom 2006.1, PANTHER 14.1, Prosite 2019_01, and Hamap 2019_01 using interproscan 5.39–77 [33]. The conserved domain database [34] at NCBI was screened online with a conserved domain search (CD-search). Potential (trans)membrane proteins were predicted online with Phobius [35]. Specific repeat domains were assessed using hmmsearch [36] on different hmm profiles (Ankyrin repeat, BTB/POZ domain, CASC3/Barentsz eIF4AIII binding, Collagen triple helix repeat, DUF3420, DUF3447, F-box, Ankyrin repeats, MORN repeat, Pentapeptide repeats). One more gene was added to the viral genome after proteomics study (see below). These results were manually integrated to improve the functional annotation. Protein structure models of remaining “hypothetical proteins” were predicted using a local instance of alphafold 2 [37] and submitted to foldseek [38] (Evalue <0.1, proba >0.9, query coverage and subject coverage >60%) to find structural homologs. All protein functions found by structural homology are indicated in orange in Table S1. One transfer RNA isoleucine was predicted using tRNA-scan-SE [39]. No ribosomal RNA was found using RNAmmer 1.2 [40]. All predicted proteins were submitted to “The MYR Predictor” web server (https://mendel.imp.ac.at/myristate) to detect myristoylation sites, then confirmed by the “Myristoylator” web server [41]. Coding and intergenic regions were extracted using Artemis software [42].

All predicted proteins were compared to the faustovirus E12 predicted proteins using blastp (Evalue 1e-5) and a reciprocal best-hit method.

### Metagenomic datasets analyses

The methods used to generate Fig. S9, Table S2, and Table S3 are described in their respective legends.

The virus genome and transpoviron sequences were masked using the dust algorithm from Meme suite version 5.5.6 [43] with default parameters and then submitted to PebbleScout [44] with default options to screen all metagenomic and metatranscriptomic runs released in a public sequence read archive (SRA) before the end of 2021. Only results with a query coverage greater than 1% were considered.

### Phylogeny

Different datasets and reference genomes were used (detailed list in Table S4): metagenome-assembled viral genomes described by Karki et al. [45] and Rigou et al. [46] and reference genomes referred to by their NCBI accession number. Marker genes were extracted using ncldv_markersearch 1.1 (available at github.com/faylward/ncldv_markersearch) and were already used for metagenomics analysis [45, 47] with the 9 possible markers (nucleocytoplasmic large DNA virus [NCLDV] major capsid protein, DEAD/SNF2-like helicase, DNA-directed RNA polymerase beta subunit, DNA-directed RNA polymerase alpha subunit, DNA polymerase family B, transcription initiation factor IIB, DNA topoisomerase II, packaging ATPase, and poxvirus viral late transcription factor 3 [VLTF3]) and aligned with Clustal Omega 1.2.4 [48]. The maximum likelihood phylogenetic tree was computed using IQ-TREE 1.6.12 [49] with the “-m TEST” model finder option and 5000 ultrafast bootstraps. The final tree representation was achieved on iTOL version 7 [50].

### Motifs and promoters

With the coding density of the viral genome being high, we focused on sequences surrounding the start codon (±100 nucleotides [nt]) instead of only intergenic regions to search for potential promoters. We first extracted the corresponding fragment for each predicted protein and submitted it to meme-chip [51] with the option -ccut 200. The same method was applied to the sequence surrounding the stop codon.

Moreover, fuzznuc and shuffleseq from the EMBOSS suite 6.6.0.0 [52] were used to search for promoters described in other NCLDVs (AAAATTGA, TATATA, and TATTT) and their corresponding shuffled sequences over the complete genomic sequence of the virus and transpoviron. The frequency of each motif was then reported in regions±100 nt around each start codon.

All motifs at least partially found in a noncoding region were counted as intergenic.

### Transpoviron analysis

In addition to the general annotation described above, the transpoviron was subjected to the following analyses. The 5′ terminal inverted repeat (TIR) sequence of pltv was isolated and compared to each matv, mvtv, and lvtv 5′ TIR using the Needleman-Wunch global alignment algorithm (EMBOSS suite). The percentage of identical nucleotides was computed for each alignment. Tandem repeats were searched on the Tandem repeat finder web server [53]. Paralogous proteins were searched using blastp (Evalue 1e^−5^) and all 12 of the predicted proteins were submitted to HHPred server [54].

### Exploring potential insertion sites

Three different methods were used to infer the transpoviron insertion sites in the viral genome: structural variations detection software (ONT PromethION reads, map and blast ONT PromethION reads, and split pairs [Illumina pair-end reads]).

The structural variations were searched as follow. All ONT PromethION reads were mapped to pacmanvirus lostcity and its transpoviron using ngmlr [55] with the *-x ont* option and its default parameters. Sniffles [55], rMETL [56], and nanoSV [57] were then run independently with their respective default parameters on the resulting mapping. The VCF files produced were analyzed using VCF-Explorer [58]. Only Sniffles detected the first structural variation confirmed by amplicon sequencing and was retained for this method.

For the second method, ONT PromethION reads were selected by mapping to the transpoviron alone using minimap 2.17 with the *-ax map-ont* option and default parameters. Coordinates of the mapping site as well as the part of the read mapped were reported. The mapped reads were then selected and aligned to the viral genome using blastn and its default parameters. Coordinates of the mapping site and the part of the read mapped were reported. The results were manually analyzed in search of virus and transpoviron sites that preserved the continuity of the mapped reads.

The last method used was based on the paired-end feature of Illumina reads and the fact that each pair was sequenced from a short fragment (between 200 and 300 bp long). All reads were mapped on the virus and transpoviron with bowtie 2.3.4.1 and the *—very-sensitive* option. Mapped reads were then filtered to only conserve 2760 perfectly matching pairs with each member on a different genomic sequence (broken mate pairs). The highest mapping depth peaks should correspond to the most probable insertion sites.

### Mass spectrometry–based characterization of virion proteome

Proteins from two independent clones of filtered virions were solubilized in Laemmli buffer and heated for 10 minutes at 95°C. They were then stacked in the top of 4%–12% NuPAGE gel (Invitrogen) and stained with Coomassie blue R-250 (Bio-Rad) before in-gel digestion using modified trypsin (Promega) as previously described [59]. The resulting peptides were analyzed by online nanoliquid chromatography coupled to tandem mass spectrometry (MS/MS) (Ultimate 3000 RSLCnano and Q-Exactive HF, Thermo Fisher Scientific) using a 120-minute gradient. For this purpose, the peptides were sampled on a precolumn (300 μm × 5 mm PepMap C18, Thermo Scientific) and separated in a 75 μm × 250 mm C18 column (Reprosil-Pur 120 C18-AQ, 1.9 μm, Dr. Maisch). Two analytical replicates were analyzed per sample. The MS and MS/MS data were acquired using Xcalibur 2.8 (Thermo Fisher Scientific).

Peptides and proteins were identified by Mascot (version 2.8.0, Matrix Science) through concomitant searches against the pacmanvirus lostcity database (474 sequences), the pltv database (12 sequences), the *A. castellanii* nuclear and mitochondrial databases (17 625 and 40 sequences, respectively), and a homemade database containing the sequences of classical contaminant proteins found in proteomic analyses (human keratins, trypsin; 126 sequences). Trypsin/P was chosen as the enzyme and two missed cleavages were allowed. Precursor and fragment mass error tolerances were set at at 10 and 20 ppm, respectively. Peptide modifications allowed during the search were carbamidomethyl (C; fixed), acetyl (protein N-term, variable), and oxidation (M, variable). Proline software [60] version 2.2.0 was used for the compilation, grouping, and filtering of the results (conservation of rank 1 peptides, peptide length ≥6 amino acids, false discovery rate of peptide-spectrum-match identifications <1% [61], and minimum of one specific peptide per identified protein group). MS data have been deposited to the ProteomeXchange Consortium via the PRIDE partner repository [62]. Proline was then used to perform an MS1 label-free quantification of the identified protein groups based on razor and specific peptides. Intensity-based absolute quantification (iBAQ) [63] values were then calculated for each protein group. The copy number of each protein in each analyzed sample was estimated by dividing the iBAQ value of each protein by that of the major capsid protein (PLN_223) before multiplying by 9240, which is the number of major capsid proteins per virion. The estimated copy numbers per clone were calculated as the average of the estimated copy numbers per analytical replicate, and the final estimated copy numbers in the pacmanvirus lostcity virions were calculated as the average of the estimated copy numbers per clone. Only proteins with an estimated copy number of at least one in the particle were further considered.

## Results

The sample collected from the Lost City hydrothermal field was put into cultures of *A. castellanii* as previously described for other giant viruses [64]. After several rounds of amplification, viral particles were collected and enriched by centrifugation or purified on sucrose gradients for various subsequent characterizations. Two biologically identical clones selected from the mixed population of viruses were grown in parallel for genome sequencing.

### Virus morphological features and replication cycle

The icosahedral virion is about 200 nm in diameter (Fig. 1A and B), exhibiting an electron-dense core, an inner membrane, and a capsid shell with a well-defined capsomer organization that can unfold in damaged particles, producing a characteristic “pacman-like” silhouette (Fig. 1C).

As described for pacmanvirus A23 [19], the replication cycle of pacmanvirus lostcity is achieved in 6 to 8 hours in *A. castellanii*, without evidence of any nuclear phase, suggesting a strictly cytoplasmic replication process. We noticed a transient change in shape of the core of pacmanvirus lostcity particles from 15 to 30 minutes postinfection (Fig. 1D) reminiscent of the conformational change observed in the phylogenetically related tornadovirus [65].

### Pacmanvirus lostcity genome structure and gene content

The longest genomic contig was obtained using the ONT PromethION–driven assembly strategy combining long and short reads. The genome is a linear double-stranded DNA molecule (33% [G + C] of 395 708 bp flanked by TIRs of about 1 kb, a structure shared within the extended family *Asfarviridae* (Table 1). The assembly graph (Fig. S1) showed numerous small variations between the positions 24 800 and 26 300 bp, leading to alternative genomic configurations for this region. This segment was then submitted to extensive PCR amplification and amplicon sequencing and the consensus sequence was retained as the main genomic structure.

**Table 1:** General characteristics of different members of the extended family *Asfarviridae.*

	**ASFV (BA71V)**	**Faustovirus (E12)**	**Kaumoebavirus**	**Tornadovirus**	**Pacmanvirus A23**	**Pacmanvirus S19**	**lupus**	**lostcity**
GenBank ID	NC_001659	KJ614390	NC_034249	LC801470	NC_034383	MZ440852	OQ411603	OK087531
Culture	Macrophages	*V. vermiformis*	*V. vermiformis*	*A.. castellanii*	*A. castellanii*	*A. castellanii*	*A. castellanii*	*A. castellanii*
Diameter (nm)	175–215	200	250	200–250	175	ND	200	150–200
Genome structure	Linear/TIR	Linear/TIR	Linear/TIR	ND^*^	Linear	Linear	Linear/TIR	Linear/TIR
Genome length (Kbp)	170	466	350	380	395	419	408	395
CDS	152	492	429	463	465	444	506^*^	473
% Coding	88,7	87,4	80	88,6	89,4	86,2	ND^*^	89,1
GC%	40,5	37	44,6	38,8	33,6	33,2	41	33,4
tRNA	0	0	0	2(Ile)	1 (Ile)	1 (Ile)	ND^*^	1 (Ile)
MCP exons	1	13/17	5	ND	1	1	ND^*^	1

ND, not or poorly defined.

^*^ From Alempic et al. [6]

The genome of pacmanvirus lostcity shares 95.2% identity (over 88.5% of the genome) and 87.6% identity (over 69.2% of the genome) with pacmanvirus A23 and S19, respectively. No TIRs were reported for the two previously sequenced pacmanviruses, probably overlooked by a less complete sequencing/assembly strategy. S19 presents the longest genome with 419 kb but exhibits a smaller number of predicted protein-coding genes (444) and a lower coding density (86.2%) compared to A23 (395 kb, 465 CoDing Sequence (CDS), 89.4% coding density) and lostcity (395 kb, 473 CDS, 89.1% coding density).

The position of pacmanvirus lostcity within the extended family *Asfarviridae* was estimated using a phylogenetic reconstruction based on nine marker genes and the largest possible number of representatives, including environmental assemblies (Fig. 2). As expected, pacmanvirus lostcity grouped with the other pacmanvirus isolates, with its closest relative being A23.

Among the 473 predicted proteins encoded by pacmanvirus lostcity, 409 had their best NCBI database matches in pacmanvirus A23, 52 in pacmanvirus S19, and three in other viruses. Eight were classified as orphan open reading frames (ORFs) or ORFans (Table S1). One ORF, PLN_356, is absent from other pacmanviruses but similar to a eukaryotic protein and contains an N-myristoyl transferase domain. A subsequent search revealed that at least four proteins from pacmanvirus lostcity exhibit a myristoylation site (PLN_184, PLN_269, PLN_411, PLN_415). These proteins are conserved in all pacmanviruses. A homology search based on 3D-structure predictions revealed the presence of two potential resolvases (PLN_245 and PLN_169) shared by the 3 pacmanviruses.

The TIRs encode two predicted hypothetical (ORFan) proteins each: PLN_1 and PLN_3, identical to PLN_473 and PLN_472 respectively.

The complete genomic sequence was scanned for late and early promoters 100 nt before the start codon of each predicted ORF and revealed the presence of an ASFV-like late promoter motif “TATATA” [66] for 165 ORFs and the mimivirus-like early promoter motif “AAAATTGA” [67] in the noncoding region or straddling the start codon of 209 ORFs and compared with faustovirus, kaumoebavirus, mimivirus, and pandoravirus dulcis genes, also scanned for the early promoter motif (Fig. S2A). With use of the same method, 200 nt around each stop codon revealed a “WWTTTATTTTTTAWW” motif with a notable enrichment at the 3′ end of 221 ORFs. Its statistical overrepresentation and preferential localization (Fig. S2B) suggest involvement in transcription termination or messenger RNA (mRNA) maturation.

Metagenomic and metatranscriptomic SRA public datasets were then searched for pacmanvirus sequences and identified reads covering from 1.09% to 10.27% of the genome were found in 21 SRA datasets from 14 soil biosamples.

### Analysis of two unexpected selfish genetic elements

During DNA purification, agarose gel migration revealed an additional band at 7 kb (Fig. S3) and a faint band around 2 kb for the highest DNA concentrations (>250 ng).

The assembly of the 7-kb DNA band was unsuccessful using a *de novo* method on three Illumina raw datasets but was achieved during the ONT PromethION–driven assembly process using all Illumina reads not mapped to the viral genome.

The 7-kb band was assembled as a linear 7244-bp double-stranded DNA molecule (38% [G + C]) with short TIRs of about 150 nt.

The similar average sequencing coverage depth of the 7-kb molecule and the main viral genome (7542 and 7659, respectively) suggests that they are loaded in equimolecular proportions in the pacmanvirus lostcity particles. The linearity of the 7-kb molecule and its length and sequence were confirmed by PCR and independent sequencing after extraction from agarose gel.

The 7-kb contig was predicted to encode 12 proteins. Sequence similarity revealed a probable helix-turn-helix (HTH) domain in pltv_1, pltv_4, and pltv_6, supported by alphafold 2 [37] models (Fig. S4). The pltv_3 ORF is predicted as a membrane protein with a signal peptide and a central transmembrane domain. The longest predicted protein, pltv_5, is closely related to helicases by its sequence and predicted structure. Given its 7 kb size, the presence of TIR, and its similarity in gene content and organization with the previously described transpovirons (Fig. 3), this pacmanvirus lostcity mobile genetic element found associated with a member of the order *Asfuvirales* expands the occurrence of transpoviron outside the *Mimiviridae* family (Fig. S5). Accordingly, we coined it “pacmanvirus lostcity transpoviron”, abbreviated as pltv.

As expected from the phylogenetic divergence of its host virus with the *Mimiviridae*, pltv exhibits some differences with the transpovirons isolated from megavirus vitis (mvtv), moumouvirus australiensis (matv) and mimivirus lentille (lvtv) (Fig. 3). Pltv is lacking the internal tandem repeat found in other transpoviron, its TIRs are less than half the size of the one of matv and also the most divergent (17% of identical nucleotides with matv, 15% with mvtv and 13% with lvtv), although *Mimiviridae* associated transpovirons share more than 50% identical nucleotides at their extremities [24]. Pltv shows a higher coding density and a greater number of predicted genes (83.2%, 12 genes; 69.1%, 6 genes for lvtv; 72.9%, 7 genes for mvtv; 75.6%, 8 genes for matv). Late and early viral promoter signatures were found in intergenic regions upstream of 6 genes (pltv_1, pltv_2 and pltv_4 to pltv_7) and of 2 genes (pltv_5 and pltv_10), respectively.

The search for the pltv sequence in metagenomes successfully identified reads covering 1.36 to 4.21% of pltv sequence in 3 datasets from 3 soil biosamples, one of them containing reads of both pacmanvirus and the transpoviron (SAMN23342724), thus expanding the occurrence of transpoviron associated with pacmanviruses in environments different from the marine thermal vent.

In contrast with the abundant and reproducible transpoviron-like 7 kb band, a faint 2 kb band visible only at high DNA concentration proved more difficult to characterize. A 1944 nt long contig (30% G + C) was assembled from the Illumina dataset of only one of the two pacmanvirus lostcity clone.

**Figure 4:**
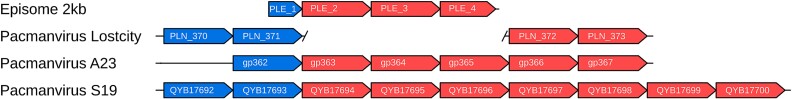
Gene content, organization, and putative origin of the 2 kb episome*.* Pacmanvirus lostcity 2 kb episome (PLE) encodes 3 homologous ORFs (PLE_2, PLE_3, PLE_4) and a truncated C-terminal part of the PNL_371 protein (PLE_1). The breach of collinearity observed in the pacmanvirus lostcity genome suggests that PLE originated from an excision in this region conserved in the other pacmanviruses. Homologous genes are colored the same.

Mapping all the reads of Illumina datasets (including both clonal and non-clonal datasets) resulted in an average coverage depth value of 11, well below the one observed for the virus (7659). Moreover, the mapped reads originated from one clonal and the non-clonal dataset and none from the other clone. This result strongly suggests the presence of a 2 kb episome in low copy number within the pacmanvirus lostcity population. We found a highly similar (99% identical) 2 kb sequence integrated in the published genome of pacmanvirus A23 (NC_034383.1, positions 315 209–317 152). A similar sequence is also partially present (82%) in pacmanvirus S19 (MZ440852.1, positions 324 927–326 410) with 77% of identity. Pacmanvirus lostcity 2 kb episome (PLE) encodes three complete predicted proteins of unknown function (PLE_2, PLE_3, PLE_4) homologous to each other (from 25% to 41.7% identical residues) preceded by an early promoter AAAATTGA. Homologs of these three genes are found in pacmanvirus A23 and S19 at the center of a colinear region between these two strains (Fig. 4), and absent from the corresponding region in pacmanvirus lostcity, as if the 2 kb episome was excised from the main viral genome. This hypothesis is further supported by the fact that the short 5′ terminal ORF (PLE_1) in the episome corresponds to the truncated 32 C-terminal amino acids of PLN_371, at the left boundary of a pacmanvirus lostcity genome colinear region with that of the other pacmanviruses (Fig. 4). Over the 4 genes, only PLE_4 is not followed by the potential transcription termination signal defined earlier. This signal is still present in the genome of pacmanvirus A23 next to the complete homologous gene.

The three PLE ORFs (PLE_2–PLE_4) genes are likely the results of ancestral duplication events in the pacmanvirus genomes, further suggested by their presence in 5 and 7 copies in pacmanvirus A23 (positions 315 402–318 459) and pacmanvirus S19 (positions 325 007–329 221), respectively (Fig. 4). The truncated PLE_1 ORF and the corresponding PLN_371 correspond to zinc finger C2H2-type domain containing proteins found in multiple copies (at least 7) along the pacmanvirus lostcity genome.

### Genomic interactions and alternative models

**Figure 5:**
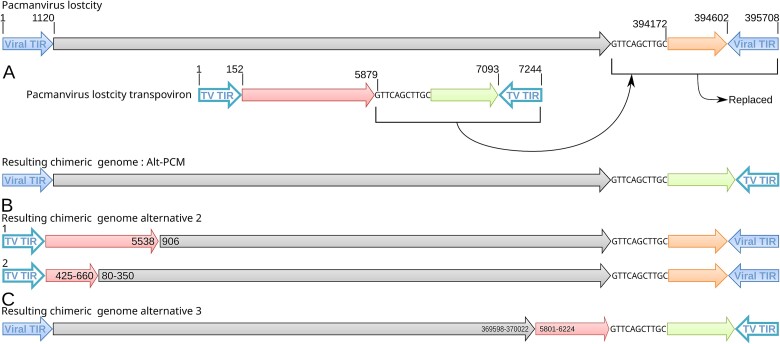
Partial integration of pltv into pacmanvirus lostcity genome*.* (A) Model of the interaction between the genomes of pltv and pacmanvirus lostcity virus leading to the virus/pltv chimeric alternative models: Alt-PCM. (B) and (C) are less represented alternative models, inferred from the mapping coverage of different insertion sites.

During the Illumina-driven assembly, an alternative genomic structure (Alt-PCM) was observed connecting most of the viral genome (positions 1–394 172) with a 3′ end part of the transpoviron (positions 5879–7244) (Fig. 5A). The two fragments overlap by 10 bp at their respective ends with the unique GTTCAGCTTGC sequence. This structure was confirmed by 19 PCR runs using primers selected on both sides of the overlapping region and producing fragments of about 1 kb. Thus, the resulting sequence of Alt-PCM encompasses one TIR from each entity.

To further investigate other potential insertion sites of the transpoviron into the viral genome, we used three different methods based on short and long reads mapping analysis. First, 114 622 ONT PromethION reads were mapped on the virus and transpoviron genome sequences and used to search for different genome organizations. In addition to the Alt-PCM already described, a second structure (Fig. 5B1) was identified connecting a major 5′ part of the pltv transpoviron (positions 1–5538) to a major 3′ end part of the viral genome (positions 906–395 708). Then, the 92 ONT PromethION reads mapping onto the transpoviron were selected and aligned to the virus genome. The junction between the two fragments is covered by 23 ONT PromethION reads. Manual investigations showed that Alt-PCM is the most represented structure. Additional less-covered structures (<10 ONT PromethION reads) were not considered. A final approach relied on the pairing properties of the Illumina reads and led to the identification of three main structures combining viral and transpoviron fragments. Again, 942 read pairs linked the viral genome down in 3′ to the transpoviron region, corresponding to the Alt-PCM structure, and 523 Illumina pairs linked the viral genomic region to the transpoviron corresponding to Fig. 5C. In the last pair (Fig. 5B2) the viral genome (80–350) was linked to pltv (425–660) by 156 read pairs. In both cases, the orientation of the reads was consistent with continuity between the viral and the transpoviron fragments. Only the three main sites are reported here but a lot of potential minor sites could be identified all along the virus and transpoviron sequences (Fig. S6).

In the experimentally confirmed Alt-PCM model, the genes pltv_9 in the transpoviron and PLN_471 in pacmanvirus were partially truncated leading to an increased ORF length for pltv_9 (pltv_9i, Fig.S7).

Altogether, these converging results strongly suggest the occurrence of recombination between the chromosomes of pacmanvirus and transpoviron. This process could involve the action of the two predicted resolvases (PLN_245 and PLN_169) and may take place at several insertion sites.

### Virion protein content

Mass spectrometry-based proteomic characterization of filtered virions from two independent clones identified 119 viral and 4 transpoviron proteins (pltv_1, pltv_2, pltv_3, and pltv_4) with an occupancy of at least one molecule per particle, as estimated using the intensity-based absolute quantification metrics [63] and the number of Major Capsid Protein (PLN_223) copies as reference (see method and Table S1). As usual for viruses, most of these virion proteins have no predicted function. Among the most abundant in the particle, we found two conserved *Asfarviridae* polyproteins (PLN_269, ranked 3^rd^ and PLN_270 ranked 4^th^) and a predicted cysteine protease (PLN_365 ranked 58^th^). As expected from a virus replicating in the cytoplasm, a complete transcription machinery is packaged in the virion, including multiple subunits of the DNA-directed RNA polymerase, a polyA polymerase, a mRNA capping enzyme, and two early transcription factors (PLN_250 and PLN_322) (Table S1). Conversely, only three proteins involved in nucleotide synthesis or replication were detected among the 18 predicted in pacmanvirus genome.

The virion also carries at least four proteins related to DNA repair (UV-damage endonuclease, flap endonuclease 1, AP endonuclease 2, DNA polymerase family X), 11 proteins belonging to the Viral metabolism/Protein modification category, and two proteins classified among the nucleic acid restriction/modification category (Table S1). A tail fiber-like protein containing a pectate lyase homologous domain (PLN_21, ranked 65^th^) and a tail spike-like protein containing a complement component 1q homologous domain (PLN_19, ranked 68^th^) were predicted to adopt different trimeric beta-helix folds (Fig. S8) and were present in 34 copies and 31 copies respectively.

The virion protein content was compared to the available faustovirus particle proteome. Most of the 59 homologous pairs correspond to hypothetical proteins but the transcription and transcript maturation machineries are present in both virions, strongly supporting a fully cytoplasmic infectious cycle. We also identified one of the two polyproteins and the thioredoxin in both proteomes (Table S1). Except for PLN_342 (hypothetical protein), 58 pacmanvirus lostcity virion proteins have no homolog in faustovirus, and are conserved in the other pacmanviruses and thus not specifically linked to the Lost City environmental conditions.

### Metagenomic datasets analysis

To provide more ecological context for the results above, the viral and eukaryotic compositions of Lost City metagenomes assembled from samples of venting fluids (PRJNA779602) and chimney biofilms (PRJNA1074139) were analyzed with multiple approaches.

Each dataset was compared to a custom database containing pacmanvirus lostcity, its transpoviron, the 2 kb episome, selected giant viruses and transpovirons using read mapping and a highly stringent filtering method. None of the pacmanvirus and companion sequences were found in the Lost City fluid or chimney metagenomes. Instead, specific sequences of 9 viruses belonging to the phylum *Nucleocytoviricota* were identified, suggesting they were more abundant than pacmanvirus lostcity (Table S2).

All reads were compared to an rRNA eukaryotic reference database. *Acanthamoebidae* were identified in less than 500 reads, but not the laboratory host used to isolate pacmanvirus (*A. castellanii*) (Fig. S9). In addition, taxonomic classification of assembled metagenomic contigs shows that *Acanthamoeba* contig coverages (Table S3) were somewhat greater in chimney grab samples (1–9 TPM) than in venting fluid samples (0–5 TPM). The chimney grab sample (LC02854) used for the virus cultivation experiments reported here was collected from the Marker 2 venting location. No metagenomes were recovered from this sample, but among venting fluid samples, Marker 2 fluids contained the highest coverage *Acanthamoeba* contigs. Among chimney grab samples, *Acanthamoeba* contigs were most abundant at Marker 6, Marker 8, and a vein of small carbonate deposits growing out of bedrock. Three *Acanthamoeba* contigs, in particular, stood out with the highest coverage (~3–4 TPM each) of all individual contigs. These contigs encode protein sequences with ~46% identities to uncharacterized proteins in the *A. castellanii* genome (XP_004338419).

## Discussion

### Pacmanviruses are ubiquitous in the environment

The first two pacmanviruses were isolated in samples from Algeria [19, 20], and we recently isolated an additional one from the intestinal remains of a 27 000 years old Siberian wolf frozen in permafrost [6]. Another relative was isolated from a Japanese river [65]. The new specimen presented here comes from a sample containing a fragment of chimney of the Lost City hydrothermal vent field, close to the Mid-Atlantic ridge at more than 700 m depth. Despite the lack of evidence of the presence of pacmanvirus lostcity and its laboratory host in Lost City metagenomes, some relatives of known families of the phylum *Nucleocytoviricota*, such as *Chrysochromulina ericina* virus, CroV, or klosneuvirus for which the host is not *Acanthamoeba*, have been identified (Table S2). Pacmanviruses present a double capsid layer and may, therefore resist the standard DNA extraction protocols used for metagenomic analyses. This also illustrates the power (and bias) of virus amplification using an appropriate host such as *A. castellanii* to isolate viruses often present in minute quantities, as previously seen for mollivirus [5]. Moreover, our analyses highlight the presence of members of the Family *Acanthamoebidae*, several of which are known to host giant viruses (Fig. S9). We can therefore assume that an as yet undescribed species within this family could be the host of pacmanvirus lostcity. A broader metagenomic analysis also confirmed the presence of pacmanviruses in additional soil samples. All pacmanviruses are intimately related but distant enough to be considered a subfamily of the *Asfarviridae*. In addition to the extremely diverse locations in which they were isolated, the detection of additional members in metagenomes of marine samples collected during a brown tide bloom in New York bays [68] and in another marine metagenome [45] allows us to extrapolate that these viruses are widely distributed, as all families of the phylum *Nucleocytoviricota* [69].

### Viral genome structure and promoters

The TIR regions of pacmanvirus lostcity have only been resolved by long reads during the assembly process. The first assembly of faustoviruses [70] and kaumoebaviruses [71] led to the proposal that they had circular genomes, but it is now accepted that they are instead linear with TIRs [72, 73]. Our results now suggest that this genomic structure is shared within the extended family *Asfarviridae* comprising pacmanviruses, kaumoebaviruses and faustoviruses.

Each strand could be covalently closed to form Flip-Flop hairpin termini, as described for members of *Asfarviridae* [74], *Poxviridae* [75], and *Phycodnaviridae* [76].

The previously defined mimiviridae early promoter motif AAAATTGA, has been found in 44% of the pacmanvirus genes. This promoter, also reported in ASFV [77], is present to a lesser extent in faustovirus and kaumoebavirus, presenting a higher GC% (Table 1). It can be found in some members of the family *Iridoviridae* and is absent from members of the family *Pandoraviridae* which are GC-rich viruses (Fig. S2A). It was proposed that in the different families of the phylum *Nucleocytoviricota* the early promoters evolved from a common ancestor [78].

### Particle protein content

More than 100 viral proteins were detected in pacmanvirus lostcity purified virions. Apart from the predicted ftsJ-like methyltransferase (PLN_90) and some transcription factors (PLN_126, PLN_206, PLN_247, PLN_408), all proteins related to transcription, including two early transcription factors (PLN_250 and PLN_322), were identified. As for ASFV [77], a full transcription machinery is packaged into the virion as expected for a virus with a fully cytoplasmic infectious cycle. In addition, as described for ASFV [79], three enzymes of the viral base excision repair system were identified in purified virions. The uracil-DNA glycosylase (PLN_393), although required for the first step of the repair process, is absent, as for ASFV. The two tail fiber–like proteins PLN_19 and PLN_21 for which the best structural models were obtained as trimers show estimated copy numbers compatible with a trimer at each vertex of the viral capsid. Both contain a domain predicted to interact with the host’s cell surface components, suggesting that PLN_19 and PLN_21 could indeed be involved in host recognition during the initial step of infection [80].

Two ASFV polyproteins, pp220 and pp60, are cleaved by the virus-encoded cysteine protease S273R into p150, p37, p14, p34, and p5 and p35, p15, and p8, respectively. These polyproteins are major components of the virion [74]. Their homologs in pacmanvirus lostcity (PLN_269, PLN_270) were also found among the most abundant proteins in the viral particle, suggesting a function and behavior equivalent to those described in ASFV. Before being cleaved, pp220 is also myristoylated [81]. Such a mechanism has also been described in other viruses and is required for viral infectivity [82] and virion structure [83]. Like most viruses, except for members of the family *Mimiviridae*, ASFV lacks the N-myristoyltransferase (NMT), and the process is likely carried out by the cellular host enzyme [83]. In contrast, pacmanvirus lostcity encodes a unique viral NMT (PLN_356) and could therefore autonomously carry out the myristoylation of PLN_269 and other predicted myristoylated proteins. For instance, PLN_415, a hypothetical membrane protein with a predicted myristoylation site present in the virion, suggests the myristoylation process in these viruses could also be involved in virus/host interactions, as described for other viruses [83].

The presence of most proteins related to transcription in pacmanvirus lostcity and faustovirus [70] virions with the absence of any visible interaction and changes of the nucleus structure during the infection cycle strongly supports an entirely cytoplasmic infectious cycle. Finally, the differences in the protein content of the two virions do not seem to be linked to the specific environment in which pacmanvirus lostcity was isolated, as all proteins specific to its virion are conserved in other pacmanviruses. As obligatory parasites, the external environmental contribution to the evolution of the viruses may not be as important as the adaptation to its host, itself being adapted to its environment.

### Identification of a new transpoviron

With the pacmanvirus lostcity genome, we also characterized a 7-kb double-stranded DNA episome resembling a transpoviron that we named pltv. Despite the absence of strong sequence similarity, pltv shares most of the characteristics of transpovirons infecting members of the family *Mimiviridae*, namely: a 7-kb–long linear DNA molecule flanked by TIRs, encoding a handful of genes including a predicted helicase and a membrane protein. The zinc finger proteins previously identified in transpovirons also have their equivalent in the HTH domain–containing proteins of pltv. These two structural motifs, although different, are well known for their role in nucleic acid binding [84], and the corresponding proteins could perform similar functions [85, 86]. Four transpoviron proteins were identified in the purified pacmanvirus virions The expression of three of them appears to be governed by late promoters. The hypothetical proteins pltv_2 and pltv_4 could thus correspond to proteins from other transpovirons found in the giant virus virions and thus could play a similar role in the delivery and/or packaging of the pltv genome [24]. The predicted transcription regulator pltv_1 has no equivalent in other transpovirons. As for the membrane protein of transpovirons associated with members of the *Mimiviridae*, pltv_3 was also detected in viral particles but with no recognizable late promoter (Fig. 3). As proposed for other transpovirons [24], it could be anchored in the inner membrane of the virions. The AAAATTGA motif, associated with early expressed genes of different members of the *Nucleocytoviricota* [64, 67, 77], is also present in the pacmanvirus lostcity genome as well as upstream of the transpoviron putative helicase (pltv_5) and pltv_10, supporting the early use of the virus transcription machinery for their expression. Pltv is thus a selfish genetic element extending the concept of a transpoviron outside of the family *Mimiviridae*, suggesting that they might be associated with other families of large DNA viruses replicating in the cytoplasm. However, the lack of strong similarities between the different transpoviron sequences makes it challenging to detect them in metagenomic data, unlike virophages [87]. The intricate propagation process previously described for transpovirons associated with members of the family *Mimiviridae* [24] can now be investigated in members of the family *Asfaviridae*.

### Virus–transpoviron interaction and alternative genome structures

Initially thought to result from assembly errors, the active genomic interaction between pacmanvirus lostcity and its transpoviron was confirmed experimentally and seems to be driven by recombination. As an episome, the transpoviron is replicated through a perennial association with the virus, and both genomes are propagated during successive infections. Given the low coverage depth of reads mapping the chimeric regions of the 3 alternative genome structures with mixed TIRs (one originating from the virus, the other from pltv) (Fig. 5B and C), two hypotheses can be proposed. Either the recombination of the transpoviron with the viral genome is a low-frequency phenomenon, or, conversely, it could be relatively common but leading to noninfectious virions, thus limiting their proportion in the viral population. Both hypotheses raise questions on the role (eventually essential) of the TIR for genome replication in pacmanviruses but also for all other viruses with TIRs, associated or not with transpovirons. Even if pltv seems to be able to integrate into the viral genome through nonspecific processes, as described for transpovirons associated with members of the *Mimiviridae* [21], our results strongly suggest some preferential integration sites and a targeted mechanism (Figs 5 and S6).

### A small episome of unclear meaning

Due to its small amount, extraction and purification of the 2-kb–long episome detected on agarose gel for sequencing was not possible. However, the pacmanvirus lostcity genome assembly process resulted in an additional unconnected 2-kb–long contig. Unexpectedly, homologous sequences were found in the genomes of the two other pacmanviruses A23 and S19. The partial collinearity of the homologous region in pacmanvirus lostcity strongly suggests that a 2-kb fragment was excised from the main genome of the virus and persisted as an episome in a small proportion of the virus particle population (hence its disappearance after recloning). The low level of similarity between three homologous ORFs encoded by the episome (PLE_2, PLE_3, PLE_4) and the presence of additional duplicates in the A23 and S19 genomes (Fig. 4) suggest a rapidly evolving unstable region. Further studies will be needed to investigate if the 2-kb fragment is self-excising and what role its encoded proteins and flanking regions play in this dynamic evolutionary process.

## Supplementary Material

Pacmanvirus_lostcity_supplementalMaterial-final

Table_S1_wraf002

Table_S2_wraf002

Table_S3_wraf002

Table_S4_wraf002

## Data Availability

The pacmanvirus lostcity genome sequence as well as its associated transpoviron sequence have been deposited in GenBank under the accession numbers OK087531 and OK087532 respectively. The 2-kb episome sequence is available as supplementary Data S1. Sequencing raw data have been deposited in the SRA database under the accession number PRJNA1126213. MS data have been deposited to the ProteomeXchange Consortium with the dataset identifier PXD054858.

## References

[1] La Scola B, Audic S, Robert C et al. A giant virus in amoebae. *Science* 2003;299:2033–3. 10.1126/science.1081867 12663918

[2] Christo-Foroux E, Alempic JM, Lartigue A et al. Characterization of Mollivirus Kamchatka, the first modern representative of the proposed Molliviridae family of Giant viruses. *J Virol* 2020;94:e01997–19. 10.1128/JVI.01997-19 31996429 PMC7108836

[3] Yoshikawa G, Blanc-Mathieu R, Song C et al. Medusavirus, a novel large DNA virus discovered from hot spring water. *J Virol* 2019;93:e02130–18. 10.1128/JVI.02130-18 30728258 PMC6450098

[4] Legendre M, Bartoli J, Shmakova L et al. Thirty-thousand-year-old distant relative of giant icosahedral DNA viruses with a pandoravirus morphology. *Proc Natl Acad Sci USA* 2014;111:4274–9. 10.1073/pnas.1320670111 24591590 PMC3964051

[5] Legendre M, Lartigue A, Bertaux L et al. In-depth study of Mollivirus sibericum, a new 30,000-y-old giant virus infecting Acanthamoeba. *Proc Natl Acad Sci USA* 2015;112:E5327–35. 10.1073/pnas.1510795112 26351664 PMC4586845

[6] Alempic JM, Lartigue A, Goncharov AE et al. An update on eukaryotic viruses revived from ancient permafrost. *Viruses.* 2023;15:564. 10.3390/v15020564 36851778 PMC9958942

[7] Kelley DS, Karson JA, Blackman DK et al. An off-axis hydrothermal vent field near the mid-Atlantic ridge at 30° N. *Nature* 2001;412:145–9. 10.1038/35084000 11449263

[8] Kelley DS, Karson JA, Früh-Green GL et al. A serpentinite-hosted ecosystem: the Lost City hydrothermal field. *Science* 2005;307:1428–34. 10.1126/science.1102556 15746419

[9] Russell MJ, Hall AJ, Martin W. Serpentinization as a source of energy at the origin of life. *Geobiology* 2010;8:355–71. 10.1111/j.1472-4669.2010.00249.x 20572872

[10] López-García P, Vereshchaka A, Moreira D. Eukaryotic diversity associated with carbonates and fluid–seawater interface in Lost City hydrothermal field. *Environ Microbiol* 2007;9:546–54. 10.1111/j.1462-2920.2006.01158.x 17222152

[11] Brazelton WJ, Baross JA. Abundant transposases encoded by the metagenome of a hydrothermal chimney biofilm. *ISME J* 2009;3:1420–4. 10.1038/ismej.2009.79 19571895

[12] He T, Li H, Zhang X. Deep-sea hydrothermal vent viruses compensate for microbial metabolism in virus-host interactions. *MBio* 2017;8:e00893–17. 10.1128/mBio.00893-17 28698277 PMC5513705

[13] Lossouarn J, Dupont S, Gorlas A et al. An abyssal mobilome: viruses, plasmids and vesicles from deep-sea hydrothermal vents. *Res Microbiol* 2015;166:742–52. 10.1016/j.resmic.2015.04.001 25911507

[14] Ortmann AC, Suttle CA. High abundances of viruses in a deep-sea hydrothermal vent system indicates viral mediated microbial mortality. *Deep Sea Res Part Oceanogr Res Pap* 2005;52:1515–27. 10.1016/j.dsr.2005.04.002

[15] Anderson RE, Sogin ML, Baross JA. Evolutionary strategies of viruses, bacteria and archaea in hydrothermal vent ecosystems revealed through metagenomics. *PLoS One* 2014;9:e109696. 10.1371/journal.pone.0109696 25279954 PMC4184897

[16] Koonin EV, Dolja VV, Krupovic M et al. Global organization and proposed Megataxonomy of the virus world. *Microbiol Mol Biol Rev* 2020;84:e00061–19. 10.1128/MMBR.00061-19 32132243 PMC7062200

[17] Bäckström D, Yutin N, Jørgensen SL et al. Virus genomes from deep sea sediments expand the ocean megavirome and support independent origins of viral gigantism. *MBio* 2019;10:e02497–18. 10.1128/mBio.02497-18 30837339 PMC6401483

[18] Schulz F, Abergel C, Woyke T. Giant virus biology and diversity in the era of genome-resolved metagenomics. *Nat Rev Microbiol* 2022;20:721–36. 10.1038/s41579-022-00754-5 35902763

[19] Andreani J, Khalil JYB, Sevvana M et al. Pacmanvirus, a new giant icosahedral virus at the crossroads between Asfarviridae and Faustoviruses. *J Virol* 2017;91:e00212–7. 10.1128/JVI.00212-17 28446673 PMC5487549

[20] Geballa-Koukoulas K, Abdi S, La Scola B et al. Pacmanvirus S19, the second pacmanvirus isolated from sewage waters in Oran. *Algeria Microbiol Resour Announc* 2021;10:e00693–21. 10.1128/MRA.00693-21 34672704 PMC8530033

[21] Desnues C, La Scola B, Yutin N et al. Provirophages and transpovirons as the diverse mobilome of giant viruses. *Proc Natl Acad Sci USA* 2012;109:18078–83. 10.1073/pnas.1208835109 23071316 PMC3497776

[22] Barth ZK, Hicklin I, Thézé J et al. Genomic analysis of hyperparasitic viruses associated with entomopoxviruses. *Virus Evol* 2024;10:veae051. 10.1093/ve/veae051 39100687 PMC11296320

[23] La Scola B, Desnues C, Pagnier I et al. The virophage as a unique parasite of the giant mimivirus. *Nature* 2008;455:100–4. 10.1038/nature07218 18690211

[24] Jeudy S, Bertaux L, Alempic JM et al. Exploration of the propagation of transpovirons within Mimiviridae reveals a unique example of commensalism in the viral world. *ISME J* 2020;14:727–39. 10.1038/s41396-019-0565-y 31822788 PMC7031253

[25] Fabre E, Jeudy S, Santini S et al. Noumeavirus replication relies on a transient remote control of the host nucleus. *Nat Commun* 2017;8:15087. 10.1038/ncomms15087 28429720 PMC5413956

[26] Bankevich A, Nurk S, Antipov D et al. SPAdes: a new genome assembly algorithm and its applications to single-cell sequencing. *J Comput Biol* 2012;19:455–77. 10.1089/cmb.2012.0021 22506599 PMC3342519

[27] Langmead B, Salzberg SL. Fast gapped-read alignment with bowtie 2. *Nat Methods* 2012;9:357–9. 10.1038/nmeth.1923 22388286 PMC3322381

[28] Li H . Minimap2: pairwise alignment for nucleotide sequences. *Bioinforma Oxf Engl* 2018;34:3094–100. 10.1093/bioinformatics/bty191 PMC6137996 29750242

[29] Walker BJ, Abeel T, Shea T et al. Pilon: an integrated tool for comprehensive microbial variant detection and genome assembly improvement. *PLoS One* 2014;9:e112963. 10.1371/journal.pone.0112963 25409509 PMC4237348

[30] Kolmogorov M, Yuan J, Lin Y et al. Assembly of long, error-prone reads using repeat graphs. *Nat Biotechnol* 2019;37:540–6. 10.1038/s41587-019-0072-8 30936562

[31] Besemer J, Lomsadze A, Borodovsky M. GeneMarkS: a self-training method for prediction of gene starts in microbial genomes. Implications for finding sequence motifs in regulatory regions. *Nucleic Acids Res* 2001;29:2607–18. 10.1093/nar/29.12.2607 11410670 PMC55746

[32] Altschul SF, Gish W, Miller W et al. Basic local alignment search tool. *J Mol Biol* 1990;215:403–10. 10.1016/S0022-2836(05)80360-2 2231712

[33] Jones P, Binns D, Chang HY et al. InterProScan 5: genome-scale protein function classification. *Bioinforma Oxf Engl* 2014;30:1236–40. 10.1093/bioinformatics/btu031 PMC3998142 24451626

[34] Marchler-Bauer A, Derbyshire MK, Gonzales NR et al. CDD: NCBI’s conserved domain database. *Nucleic Acids Res* 2015;43: D222–6. 10.1093/nar/gku1221 25414356 PMC4383992

[35] Käll L, Krogh A, Sonnhammer ELL. A combined transmembrane topology and signal peptide prediction method. *J Mol Biol* 2004;338:1027–36. 10.1016/j.jmb.2004.03.016 15111065

[36] Eddy SR . Accelerated profile HMM searches. *PLoS Comput Biol* 2011;7:e1002195. 10.1371/journal.pcbi.1002195 22039361 PMC3197634

[37] Jumper J, Evans R, Pritzel A et al. Highly accurate protein structure prediction with AlphaFold. *Nature* 2021;596:583–9. 10.1038/s41586-021-03819-2 34265844 PMC8371605

[38] van Kempen M, Kim SS, Tumescheit C et al. Fast and accurate protein structure search with Foldseek. *Nat Biotechnol* 2024;42:243–6. 10.1038/s41587-023-01773-0 37156916 PMC10869269

[39] Chan PP, Lowe TM. tRNAscan-SE: searching for tRNA genes in genomic sequences. *Methods Mol Biol Clifton NJ* 2019;1962:1–14. 10.1007/978-1-4939-9173-0_1 PMC6768409 31020551

[40] Lagesen K, Hallin P, Rødland EA et al. RNAmmer: consistent and rapid annotation of ribosomal RNA genes. *Nucleic Acids Res* 2007;35:3100–8. 10.1093/nar/gkm160 17452365 PMC1888812

[41] Bologna G, Yvon C, Duvaud S et al. N-terminal myristoylation predictions by ensembles of neural networks. *Proteomics* 2004;4:1626–32. 10.1002/pmic.200300783 15174132

[42] Rutherford K, Parkhill J, Crook J et al. Artemis: sequence visualization and annotation. *Bioinforma Oxf Engl* 2000;16:944–5. 10.1093/bioinformatics/16.10.944 11120685

[43] Bailey TL, Johnson J, Grant CE et al. The MEME suite. *Nucleic Acids Res* 2015;43:W39–49. 10.1093/nar/gkv416 25953851 PMC4489269

[44] Shiryev SA, Agarwala R. Indexing and searching petabase-scale nucleotide resources. *Nat Methods* 2024;21:994–1002. 10.1038/s41592-024-02280-z 38755321 PMC11166510

[45] Karki S, Moniruzzaman M, Aylward FO. Comparative genomics and environmental distribution of large dsDNA viruses in the family Asfarviridae. *Front Microbiol* 2021;12:657471. 10.3389/fmicb.2021.657471 33790885 PMC8005611

[46] Rigou S, Santini S, Abergel C et al. Past and present giant viruses diversity explored through permafrost metagenomics. *Nat Commun* 2022;13:5853. 10.1038/s41467-022-33633-x 36207343 PMC9546926

[47] Moniruzzaman M, Martinez-Gutierrez CA, Weinheimer AR et al. Dynamic genome evolution and complex virocell metabolism of globally-distributed giant viruses. *Nat Commun* 2020;11:1710. 10.1038/s41467-020-15507-2 32249765 PMC7136201

[48] Sievers F, Wilm A, Dineen D et al. Fast, scalable generation of high-quality protein multiple sequence alignments using Clustal omega. *Mol Syst Biol* 2011;7:539. 10.1038/msb.2011.75 21988835 PMC3261699

[49] Nguyen LT, Schmidt HA, Von Haeseler A et al. IQ-TREE: a fast and effective stochastic algorithm for estimating maximum-likelihood phylogenies. *Mol Biol Evol* 2015;32:268–74. 10.1093/molbev/msu300 25371430 PMC4271533

[50] Letunic I, Bork P. Interactive tree of life (iTOL) v5: an online tool for phylogenetic tree display and annotation. *Nucleic Acids Res* 2021;49:W293–6. 10.1093/nar/gkab301 33885785 PMC8265157

[51] Machanick P, Bailey TL. MEME-ChIP: motif analysis of large DNA datasets. *Bioinformatics* 2011;27:1696–7. 10.1093/bioinformatics/btr189 21486936 PMC3106185

[52] Rice P, Longden I, Bleasby A. EMBOSS: the European molecular biology open software suite. *Trends Genet TIG* 2000;16:276–7. 10.1016/S0168-9525(00)02024-2 10827456

[53] Benson G . Tandem repeats finder: a program to analyze DNA sequences. *Nucleic Acids Res* 1999;27:573–80. 10.1093/nar/27.2.573 9862982 PMC148217

[54] Zimmermann L, Stephens A, Nam SZ et al. A completely reimplemented MPI bioinformatics toolkit with a new HHpred server at its core. *J Mol Biol* 2018;430:2237–43. 10.1016/j.jmb.2017.12.007 29258817

[55] Sedlazeck FJ, Rescheneder P, Smolka M et al. Accurate detection of complex structural variations using single molecule sequencing. *Nat Methods* 2018;15:461–8. 10.1038/s41592-018-0001-7 29713083 PMC5990442

[56] Jiang T, Liu B, Li J et al. rMETL: sensitive mobile element insertion detection with long read realignment. *Bioinformatics* 2019;35:3484–6. 10.1093/bioinformatics/btz106 30759188

[57] Cretu Stancu M, van Roosmalen MJ, Renkens I et al. Mapping and phasing of structural variation in patient genomes using nanopore sequencing. *Nat Commun* 2017;**8**:1326–38. 10.1038/s41467-017-01343-4 PMC5673902 29109544

[58] Akgün M, Demirci H. VCF-explorer: filtering and analysing whole genome VCF files. *Bioinformatics* 2017;33:3468–70. 10.1093/bioinformatics/btx422 29036499

[59] Casabona MG, Vandenbrouck Y, Attree I et al. Proteomic characterization of Pseudomonas aeruginosa PAO1 inner membrane. *Proteomics* 2013;13:2419–23. 10.1002/pmic.201200565 23744604

[60] Bouyssié D, Hesse AM, Mouton-Barbosa E et al. Proline: an efficient and user-friendly software suite for large-scale proteomics. *Bioinforma Oxf Engl* 2020;36:3148–55. 10.1093/bioinformatics/btaa118 PMC7214047 32096818

[61] Couté Y, Bruley C, Burger T. Beyond target-decoy competition: stable validation of peptide and protein identifications in mass spectrometry-based discovery proteomics. *Anal Chem* 2020;92:14898–906. 10.1021/acs.analchem.0c00328 32970414

[62] Perez-Riverol Y, Csordas A, Bai J et al. The PRIDE database and related tools and resources in 2019: improving support for quantification data. *Nucleic Acids Res* 2019;47:D442–50. 10.1093/nar/gky1106 30395289 PMC6323896

[63] Schwanhäusser B, Busse D, Li N et al. Global quantification of mammalian gene expression control. *Nature* 2011;473:337–42. 10.1038/nature10098 21593866

[64] Arslan D, Legendre M, Seltzer V et al. Distant Mimivirus relative with a larger genome highlights the fundamental features of Megaviridae. *Proc Natl Acad Sci USA* 2011;108:17486–91. 10.1073/pnas.1110889108 21987820 PMC3198346

[65] Waschestjuk D, Murata K, Takemura M. Complete genome sequence of Tornadovirus japonicus, a relative of Pacmanvirus, isolated from the Tamagawa River in Japan. *Microbiol Resour Announc* 2024;13:e00265–24. 10.1128/mra.00265-24 38860801 PMC11256800

[66] García-Escudero R, Viñuela E. Structure of African swine fever virus late promoters: requirement of a TATA sequence at the initiation region. *J Virol* 2000;74:8176–82. 10.1128/JVI.74.17.8176-8182.2000 10933729 PMC112352

[67] Suhre K, Audic S, Claverie JM. Mimivirus gene promoters exhibit an unprecedented conservation among all eukaryotes. *Proc Natl Acad Sci USA* 2005;102:14689–93. 10.1073/pnas.0506465102 16203998 PMC1239944

[68] Gann ER, Kang Y, Dyhrman ST et al. Metatranscriptome library preparation influences analyses of viral community activity during a Brown tide bloom. *Front Microbiol* 2021;12:664189. 10.3389/fmicb.2021.664189 34135876 PMC8200674

[69] Schulz F, Roux S, Paez-Espino D et al. Giant virus diversity and host interactions through global metagenomics. *Nature* 2020;578:432–6. 10.1038/s41586-020-1957-x 31968354 PMC7162819

[70] Reteno DG, Benamar S, Khalil JB et al. Faustovirus, an Asfarvirus-related new lineage of Giant viruses infecting amoebae. *J Virol* 2015;89:6585–94. 10.1128/JVI.00115-15 25878099 PMC4468488

[71] Bajrai LH, Benamar S, Azhar EI et al. Kaumoebavirus, a new virus that clusters with Faustoviruses and Asfarviridae. *Viruses* 2016;8:278. 10.3390/v8110278 27801826 PMC5127008

[72] Geballa-Koukoulas K, Boudjemaa H, Andreani J et al. Comparative genomics unveils regionalized evolution of the Faustovirus genomes. *Viruses* 2020;12:577. 10.3390/v12050577 32456325 PMC7290515

[73] Geballa-Koukoulas K, Andreani J, La Scola B et al. The Kaumoebavirus LCC10 genome reveals a unique gene strand bias among “extended Asfarviridae”. *Viruses* 2021;13:148. 10.3390/v13020148 33498382 PMC7909422

[74] Dixon LK, Chapman DAG, Netherton CL et al. African swine fever virus replication and genomics. *Virus Res* 2013;173:3–14. 10.1016/j.virusres.2012.10.020 23142553

[75] Lefkowitz EJ, Wang C, Upton C. Poxviruses: past, present and future. *Virus Res* 2006;117:105–18. 10.1016/j.virusres.2006.01.016 16503070

[76] Kang M, Dunigan DD, Van Etten JL. *Chlorovirus* : a genus of Phycodnaviridae that infects certain chlorella-like green algae. *Mol Plant Pathol* 2005;6:213–24. 10.1111/j.1364-3703.2005.00281.x 20565652

[77] Cackett G, Sýkora M, Werner F. Transcriptome view of a killer: African swine fever virus. *Biochem Soc Trans* 2020;48:1569–81. 10.1042/BST20191108 32725217 PMC7458399

[78] Oliveira GP, Andrade AC d SP, Rodrigues RAL et al. Promoter motifs in NCLDVs: an evolutionary perspective. *Viruses* 2017;9:16. 10.3390/v9010016 28117683 PMC5294985

[79] Redrejo-Rodriguez M, Rodríguez MJ, Salas JLM. Repair of viral genomes by base excision pathways: African swine fever virus as a paradigm. In: Storici F. (ed.), DNA Repair - on the Pathways to Fixing DNA Damage and Errors [Internet]. London, UK: InTech, 2011, [cited 6 August 2024]. Available from: 10.5772/23328

[80] Mitraki A, Miller S, van Raaij MJ. Review: conformation and folding of novel beta-structural elements in viral fiber proteins: the triple beta-spiral and triple beta-helix. *J Struct Biol* 2002;137:236–47. 10.1006/jsbi.2002.4447 12064949

[81] Simón-Mateo C, Andrés G, Viñuela E. Polyprotein processing in African swine fever virus: a novel gene expression strategy for a DNA virus. *EMBO J* 1993;12:2977–87. 10.1002/j.1460-2075.1993.tb05960.x 8335009 PMC413553

[82] Kräusslich HG, Hölscher C, Reuer Q et al. Myristoylation of the poliovirus polyprotein is required for proteolytic processing of the capsid and for viral infectivity. *J Virol* 1990;64:2433–6. 10.1128/jvi.64.5.2433-2436.1990 2157900 PMC249411

[83] Maurer-Stroh S, Eisenhaber F. Myristoylation of viral and bacterial proteins. *Trends Microbiol* 2004;12:178–85. 10.1016/j.tim.2004.02.006 15051068

[84] Schleif R . DNA binding by proteins. *Science* 1988;241:1182–7. 10.1126/science.2842864 2842864

[85] Aravind L, Anantharaman V, Balaji S et al. The many faces of the helix-turn-helix domain: transcription regulation and beyond. *FEMS Microbiol Rev* 2005;29:231–62. 10.1016/j.femsre.2004.12.008 15808743

[86] Laity JH, Lee BM, Wright PE. Zinc finger proteins: new insights into structural and functional diversity. *Curr Opin Struct Biol* 2001;11:39–46. 10.1016/S0959-440X(00)00167-6 11179890

[87] Roitman S, Rozenberg A, Lavy T et al. Isolation and infection cycle of a polinton-like virus virophage in an abundant marine alga. *Nat Microbiol* 2023;8:332–46. 10.1038/s41564-022-01305-7 36702941

