# Supplementary material for: Pacmanvirus isolated from the Lost City hydrothermal field extends the concept of transpoviron beyond the family Mimiviridae
Source: ISME J. 2025 Jan 10;19(1):wraf002. doi: 10.1093/ismejo/wraf002 (PMC11788076; doi:10.1093/ismejo/wraf002)
Supplement: Pacmanvirus_lostcity_supplementalMaterial-final [file pacmanvirus_lostcity_supplementalmaterial-final.pdf]

Supplemental Material

Pacmanvirus isolated from the Lost City hydrothermal field extends the concept of transpoviron beyond the family *Mimiviridae*.

Sébastien Santini, Audrey Lartigue, Jean-Marie Alempic, Yohann Couté, Lucid Belmudes, William J. Brazelton, Susan Q. Lang, Jean-Michel Claverie, Matthieu Legendre, Chantal Abergel

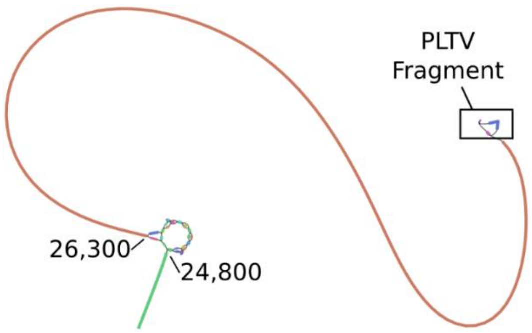

Figure S1: Graphical representation of one assembly graph of pacmanvirus lostcity
This assembly graph was obtained with Bandage *(1)*.

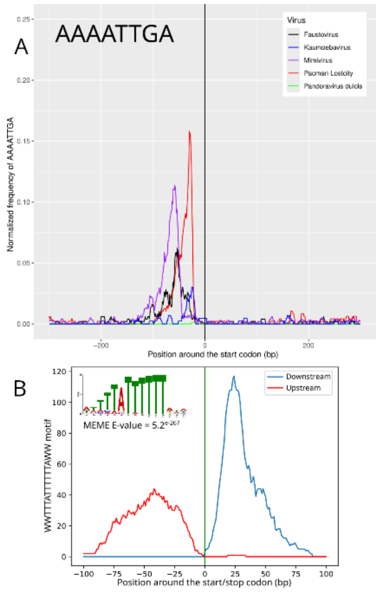

Figure S2: Early promoter and potential transcription termination signal sequence and repartitions
A) Early promoter repartition in pacmanvirus lostcity, faustovirus, kaumoebavirus, mimivirus and pandoravirus dulcis. All viruses were analyzed using the same method, described in the material and method section. The number of AAAATTGA motif at each position is normalized by the total number of ORFs. B) The logo sequence describes WWTTTATTTTTAWW, the most represented motif obtained with meme-chip *(2)* used to screen all 200nt sequences surrounding the beginning and the end of each gene with fimo *(3)*. The green line represents the stop or the start codon position regarding the downstream (blue) or the upstream (red) analysis respectively. Only forward motifs were reported.

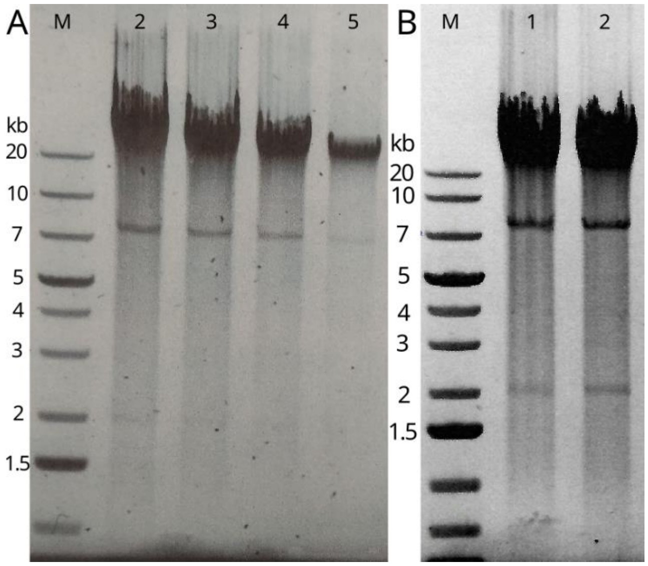

Figure S3: Agarose gel migration of pacman lostcity extracted DNA

A) Four wells were loaded with decreasing DNA quantities (1µg, 500 ng, 150ng and 125ng). Marker lengths (in kbp) correspond to "M" Lanes 1. B) Two wells were loaded with 1 and 1.5 µg of DNA extracted from the virions of pacmanvirus lostcity before cloning.

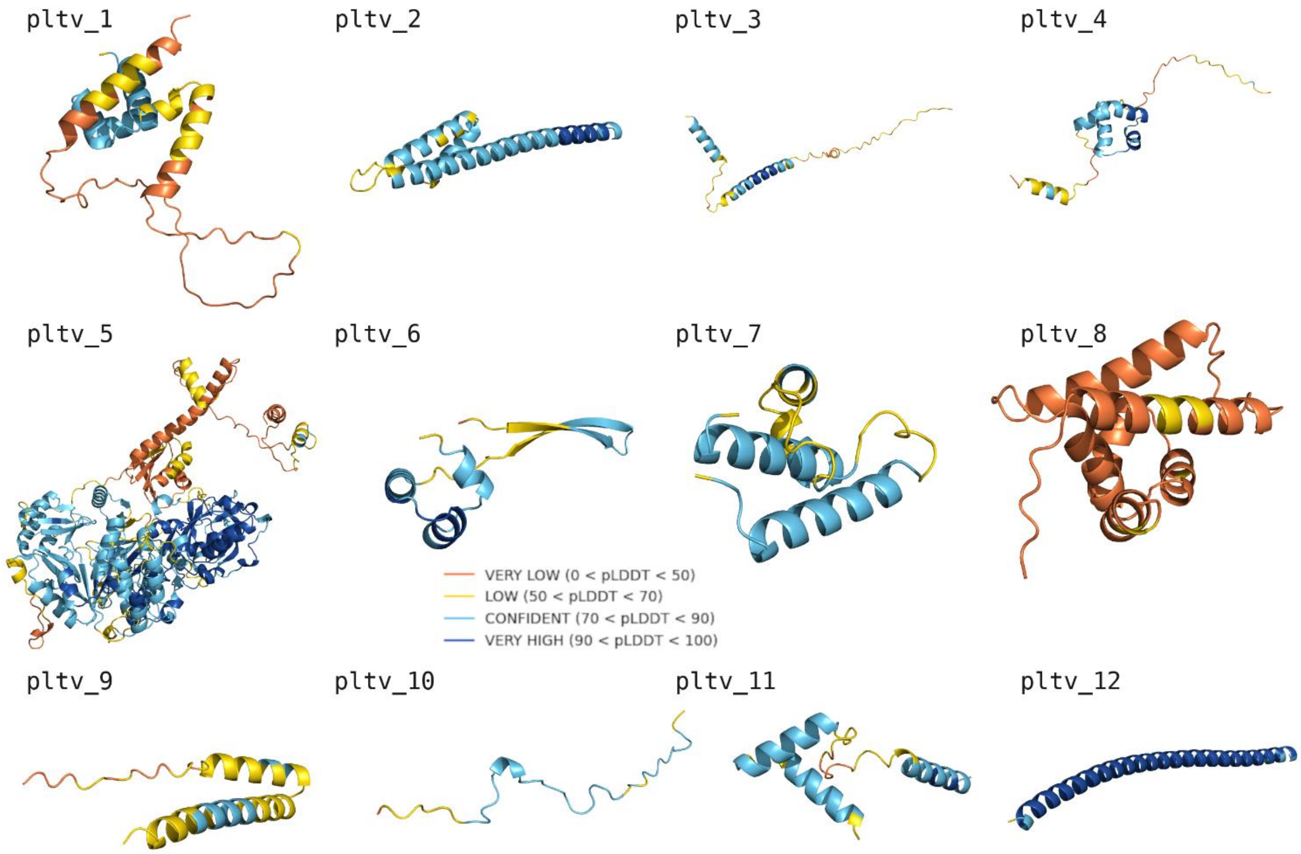

Figure S4: Structural models of pacmanvirus lostcity transpoviron predicted proteins.
All models were obtained using a local instance of alphafold 2 *(4)* with default parameters. The pLDDT value reflecting the confidence is used to color the structure from red (very low confidence) to blue (very high confidence).

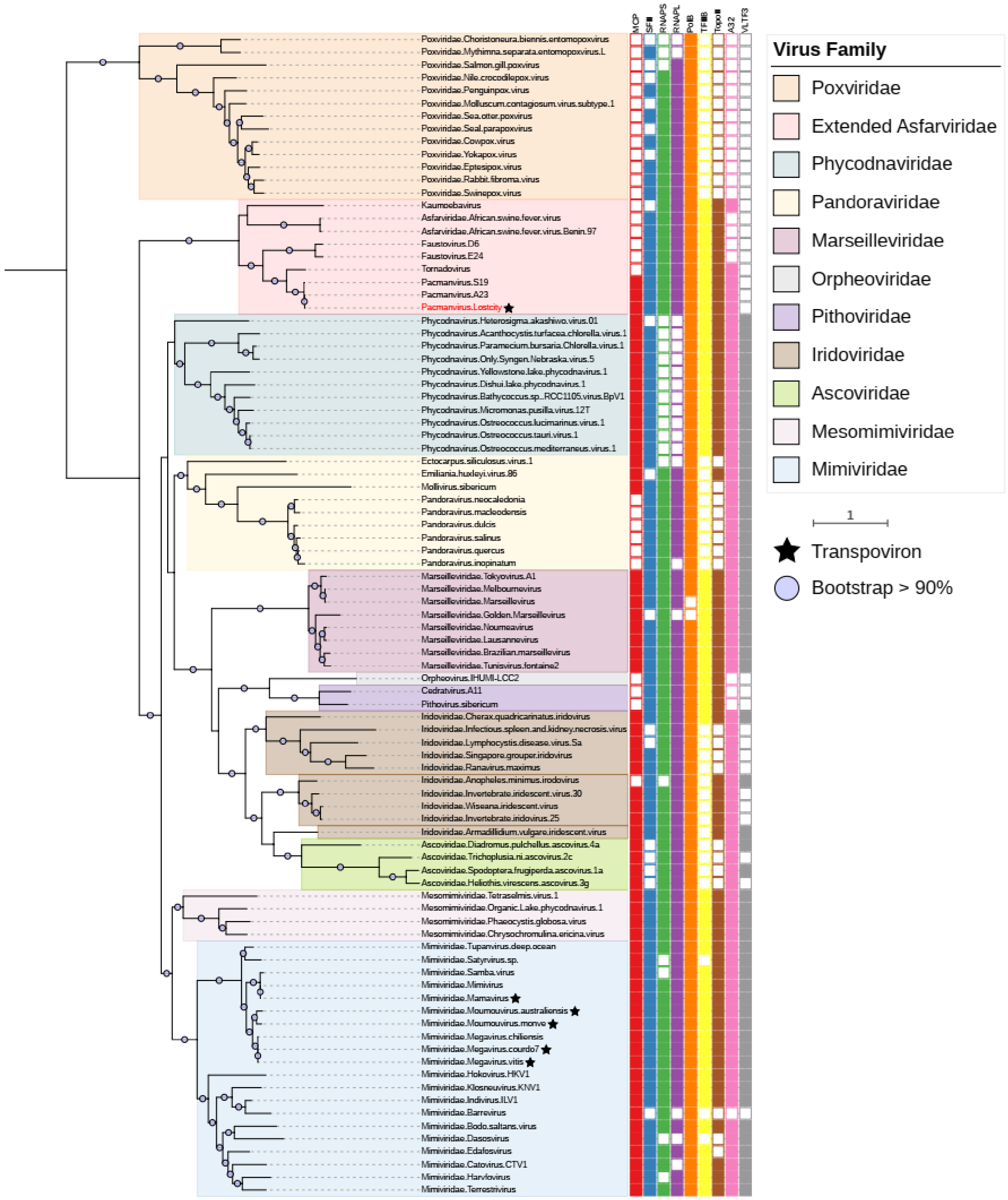

Figure S5: Phylogeny of selected viruses from the Phylum *Nucleocitoviricota*. Marker genes were extracted using ncldv_markersearch 1.1 (available at github.com/faylward/ncldv_markersearch) and already used for metagenomics analysis with the 9 possible markers (NCLDV major capsid protein, DEAD/SNF2-like helicase, DNA-directed RNA polymerase beta subunit, DNA-directed RNA

polymerase alpha subunit, DNA polymerase family B, Transcription initiation factor IIB, DNA topoisomerase II, Packaging ATPase, Poxvirus Late Transcription Factor VLTF3) and aligned with Clustal Omega v1.2.4 *(5)*. The alignment was curated with CIAlign v1.1.4 (--remove_insertions --insertion_max_size 10000 --insertion_min_flank 0) *(6)* and then submitted to IQ-TREE v1.6.12 *(7)* (-bb 5000 -bi 200 -m TEST). The phylogenetic tree obtained was annotated with iTOL v7 *(8)*. An empty square indicates that the marker was not found by ncldv_markersearch. Viruses known to be associated with a transpoviron are marked with a black star. The scale bar is displayed below the legend box. Only bootstraps greater than 90% are reported.

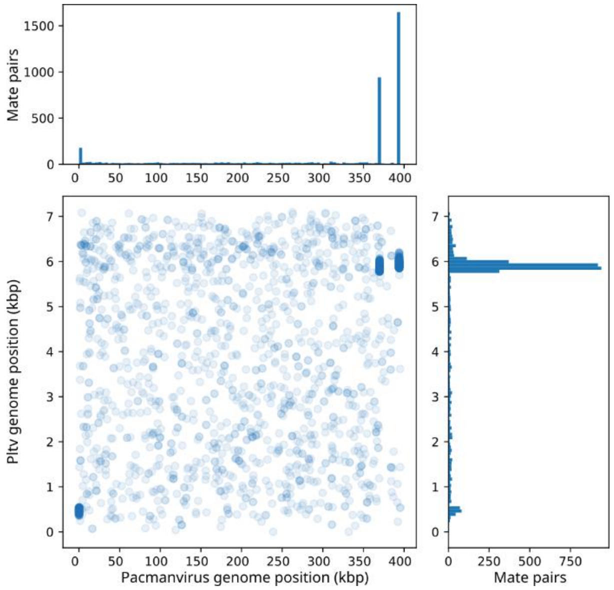

Figure S6: Shared read pairs between the pacmanvirus lostcity and pltv sequences
Read mapping was obtained as described in the methods section. Only paired reads mapping both sequences were conserved. Plot and histograms were drawn using standard python libraries.

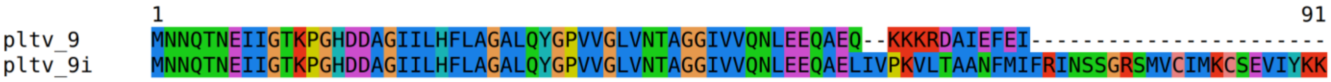

Figure S7: Alignment of the two versions of the pltv_9 protein.
Pltv_9 corresponds to the transpoviron gene and pltv_9i is the result of the recombination between the transpoviron and the virus resulting in the Alt-PCM chimeric genome model.

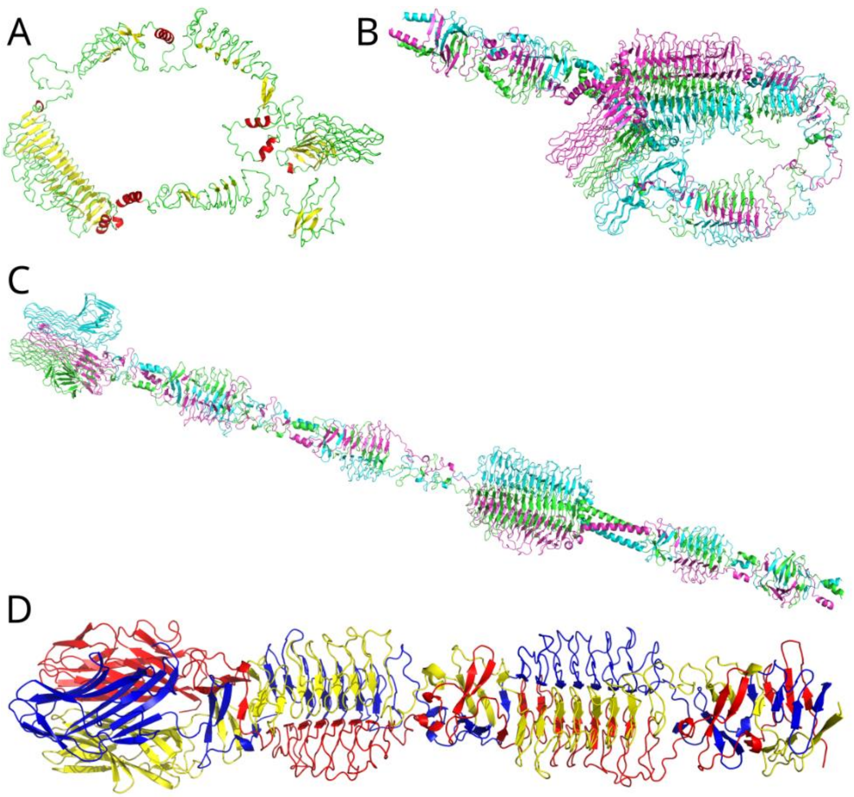

Figure S8: Alphafold tail spike-like and tail fiber-like protein models.
Tail fiber-like PLN_21 A) monomeric and B) trimeric structures as predicted using the complete sequence. C) PLN_21 trimeric structure model using fragments. The protein was manually fragmented to obtain different sequence lengths corresponding to residues: 1-500, 500-1370, 250-750, 1-660, 660-1370. Each fragment was then submitted as a homo-trimer to our alphafold *(4,9)* local instance. The best model of each prediction was used to reconstruct the final model by structural alignment, leading to the linear form of the PLN_21 trimeric structure. D) alphafold tail spike-like PLN_19 trimeric model.

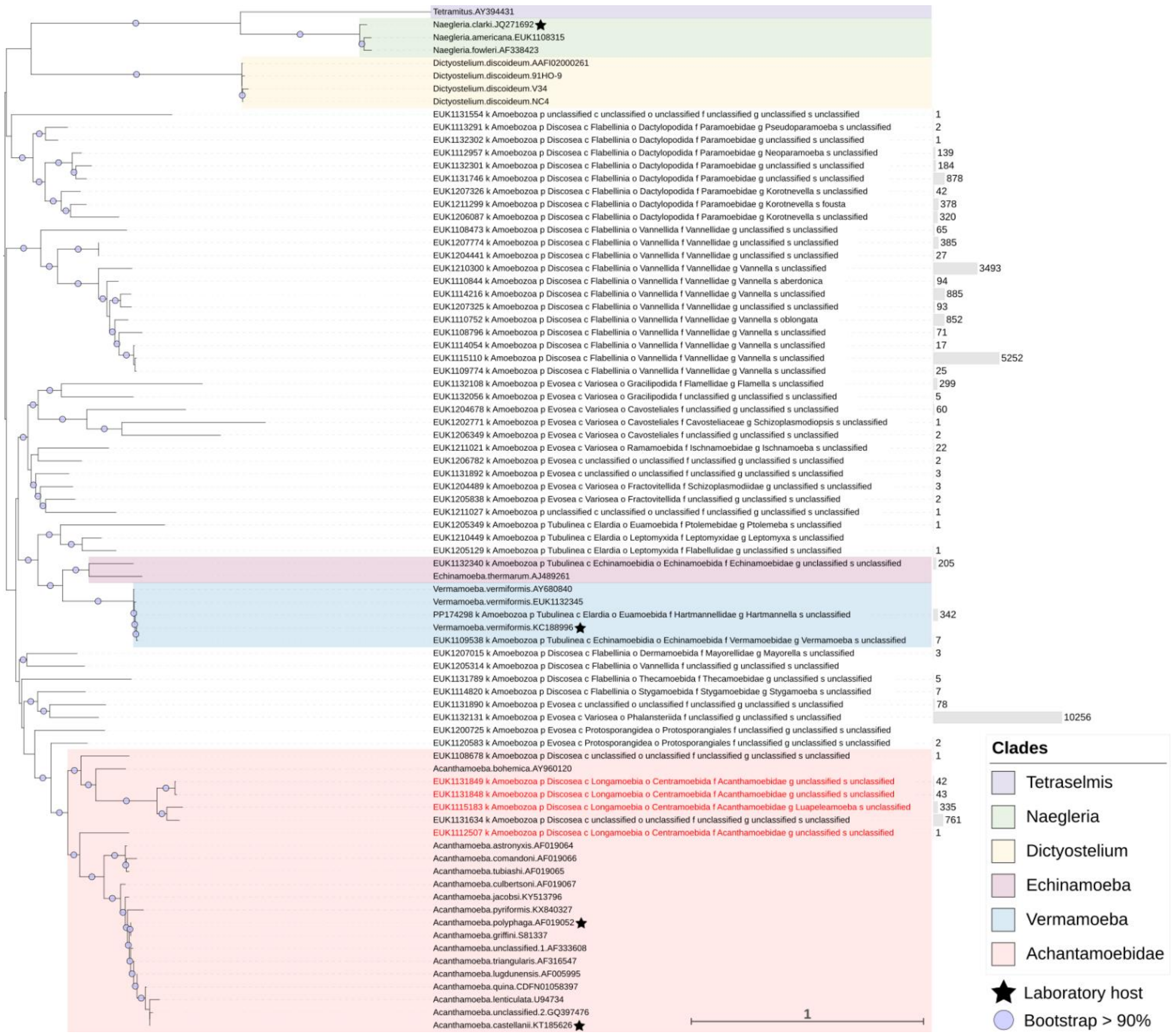

Figure S9: Phylogeny of all *Amoebozoa* found in the Lost City metagenomic datasets (PRJNA779602 and PRJNA1074139).

All reads were compared to the eukaryotic rRNA gene database EUKARYOME version General_EUK_longread_v1.9 *(10)* using blastn *(11)* (Evalue 1e-20). Only the best hits with 100% of identity over 100% of each read length were reported. The entire pair was rejected if it did not match the same target. The targeted rRNA identified as *Amoebozoa* using the EUKARYOME taxonomy as well as a selection of reference rRNA from the database were aligned using mafft v7.505 with default options. The alignment was curated with CIAlign v1.1.4 *(6)* (--remove_insertions --insertion_max_size 10000 --insertion_min_flank 0) then submitted to IQ-TREE v1.6.12 *(7)* (-bb 5000 -bi 200 -m TEST). The phylogenetic tree obtained was annotated with iTOL v7 *(8)*. *Acanthamoebidae* sequences are indicated in red and the scale bar reported below the tree. Only bootstraps greater than 90% are reported.

Table S1: Virus and transpoviron predicted proteins

Pacmanvirus and pltv predicted proteins are listed in ascending order of gene number. Proteins identified by MS-based proteomics are indicated with a grey background. Their estimated copy numbers and

corresponding rank, as described in the method section, are reported. Predicted promoters are labeled as follows: E for early, L for late, B for both early and late, N for none detected. The presence of potential transcription termination signal is indicated with an X. An orange background means a functional annotation using structural homology (alphafold 2 and foldseek *(12)* – see methods). Homologous proteins identified in the faustovirus *(13)* virion proteome are marked by a X in the last column. A star (*) indicates that the best hit was recovered using tblastn. A sharp sign (#) indicates that no tblastn hit was found in A23 or S19.

Table S2: List of viruses used for mapping of Lost City metagenomic datasets (PRJNA779602 and PRJNA1074139)

All reads were mapped to a laboratory collection of 86 viruses and transpovirons using bowtie 2 with the --very-sensitive option. Only paired reads longer than 20 bp were used to analysis. The mapped regions of each virus were extracted and submitted to dust. Remaining genome fragments longer than 64 bp (half the shortest mean of reads) were submitted to blastn against the core_nt database at the NCBI. If the percentage of identity was greater than 90%, the query coverage greater than 50%, the main hits of the same species and no other species was detected with the exact same parameters, the sequence was retained as specific enough to characterize the presence of the virus in the sample. An X in the right column indicates one of these sequences.

Table S3: Taxonomic assignment of assembled metagenomic contigs

Metagenomic reads from Lost City fluids and chimney biofilms were assembled with Megahit v1.1.1 *(14)* as previously described *(15)*. Assembled contigs were classified against the UniRef100 database using the mmseqs2 classifier *(16)*. Taxonomic classification was conducted with both the DNA sequences of whole contigs and individual predicted protein sequences. The taxonomic assignment of each contig classified as genus Acanthamoeba (with either the DNA or protein search) was reported along with its sequence coverage calculated in units of transcripts (i.e. fragments) per million (TPM), as previously described *(15)*. Similar results were achieved at the family level (*Acanthamoebidae*).

Table S4: Dataset used to generate the phylogeny described in Figure 2

The MAGs datasets are referred to as described by Karki et *al. (17)* and Rigou et *al. (18)*. The reference genomes are referred to by their NCBI accession number.

Supplementary References

1. Wick RR, Schultz MB, Zobel J, Holt KE. Bandage: interactive visualization of *de novo* genome assemblies. Bioinformatics. 2015 Oct 15;31(20):3350–2.

2. Machanick P, Bailey TL. MEME-ChIP: motif analysis of large DNA datasets. Bioinformatics. 2011 Jun 15;27(12):1696–7.

3. Grant CE, Bailey TL, Noble WS. FIMO: scanning for occurrences of a given motif. Bioinforma Oxf Engl. 2011 Apr 1;27(7):1017–8.

4. Jumper J, Evans R, Pritzel A, Green T, Figurnov M, Ronneberger O, et al. Highly accurate protein structure prediction with AlphaFold. Nature. 2021 Aug;596(7873):583–9.

5. Sievers F, Wilm A, Dineen D, Gibson TJ, Karplus K, Li W, et al. Fast, scalable generation of high-quality protein multiple sequence alignments using Clustal Omega. Mol Syst Biol. 2011 Oct 11;7:539.

6. Tumescheit C, Firth AE, Brown K. CIAlign: A highly customisable command line tool to clean, interpret and visualise multiple sequence alignments. PeerJ. 2022 Mar 15;10:e12983.

7. Nguyen LT, Schmidt HA, Von Haeseler A, Minh BQ. IQ-TREE: A Fast and Effective Stochastic Algorithm for Estimating Maximum-Likelihood Phylogenies. Mol Biol Evol. 2015 Jan;32(1):268–74.

8. Letunic I, Bork P. Interactive Tree of Life (iTOL) v6: recent updates to the phylogenetic tree display and annotation tool. Nucleic Acids Res. 2024 Jul 5;52(W1):W78–82.

9. Evans R, O'Neill M, Pritzel A, Antropova N, Senior A, Green T, et al. Protein complex prediction with AlphaFold-Multimer [Internet]. 2021 [cited 2024 Jun 24]. Available from: http://biorxiv.org/lookup/doi/10.1101/2021.10.04.463034

10. Tedersoo L, Hosseyni Moghaddam MS, Mikryukov V, Hakimzadeh A, Bahram M, Nilsson RH, et al. EUKARYOME: the rRNA gene reference database for identification of all eukaryotes. Database J Biol Databases Curation. 2024 Jun 12;2024:baae043.

11. Altschul SF, Gish W, Miller W, Myers EW, Lipman DJ. Basic local alignment search tool. J Mol Biol. 1990 Oct 5;215(3):403–10.

12. van Kempen M, Kim SS, Tumescheit C, Mirdita M, Lee J, Gilchrist CLM, et al. Fast and accurate protein structure search with Foldseek. Nat Biotechnol. 2024 Feb;42(2):243–6.

13. Reteno DG, Benamar S, Khalil JB, Andreani J, Armstrong N, Klose T, et al. Faustovirus, an Asfarvirus-Related New Lineage of Giant Viruses Infecting Amoebae. J Virol. 2015 Apr 15;89(13):6585–94.

14. Li D, Liu CM, Luo R, Sadakane K, Lam TW. MEGAHIT: an ultra-fast single-node solution for large and complex metagenomics assembly via succinct de Bruijn graph. Bioinforma Oxf Engl. 2015 May 15;31(10):1674–6.

15. Brazelton WJ, McGonigle JM, Motamedi S, Pendleton HL, Twing KI, Miller BC, et al. Metabolic Strategies Shared by Basement Residents of the Lost City Hydrothermal Field. Appl Environ Microbiol. 2022 Sep 13;88(17):e0092922.

16. Steinegger M, Söding J. MMseqs2 enables sensitive protein sequence searching for the analysis of massive data sets. Nat Biotechnol. 2017 Nov;35(11):1026–8.

17. Karki S, Moniruzzaman M, Aylward FO. Comparative Genomics and Environmental Distribution of Large dsDNA Viruses in the Family Asfarviridae. Front Microbiol. 2021 Mar 15;12:657471.

18. Rigou S, Santini S, Abergel C, Claverie JM, Legendre M. Past and present giant viruses diversity explored through permafrost metagenomics. Nat Commun. 2022 Oct 7;13(1):5853.
